# Human Colitis‐on‐Chip Model Reveals Dual Roles of Butyrate in Epithelial and Macrophage Defense Against *Candida albicans* Tissue Invasion

**DOI:** 10.1002/smll.202600074

**Published:** 2026-05-15

**Authors:** Manuel Allwang, Maximilian Wipplinger, Parastoo Akbarimoghaddam, Raquel Alonso‐Roman, Zoltan Cseresnyes, Axel Dietschmann, Valentin Wegner, Adrian Feile, Yann Bachelot, Maria Warschinke, Mohamed I. Abdelwahab Hassan, Sonnhild Mittag, Otmar Huber, Bernhard Hube, Mark S. Gresnigt, Marc Thilo Figge, Alexander S. Mosig

**Affiliations:** ^1^ Institute of Biochemistry II Jena University Hospital Jena Germany; ^2^ Cluster of Excellence Balance of the Microverse Friedrich Schiller University Jena Germany; ^3^ Applied Systems Biology Leibniz Institute for Natural Product Research and Infection Biology Hans Knöll Institute (HKI) Jena Germany; ^4^ Faculty of Biological Sciences Friedrich Schiller University Jena Germany; ^5^ Junior Research Group Adaptive Pathogenicity Strategies Leibniz Institute for Natural Product Research and Infection Biology Hans Knöll Institute (HKI) Jena Germany; ^6^ Department of Microbial Pathogenicity Mechanisms Leibniz Institute for Natural Product Research and Infection Biology Hans Knöll Institute (HKI) Jena Germany; ^7^ Institute of Microbiology Friedrich Schiller University Jena Germany

**Keywords:** IBD, infection, inflammation, microbiome, short‐chain fatty acids

## Abstract

Microbial dysbiosis in inflammatory bowel disease (IBD) reduces intestinal butyrate levels, compromising epithelial barrier integrity and enabling overgrowth of opportunistic pathogens such as *Candida albicans*. Here, we present a human immunocompetent colitis‐on‐chip model (CooC) that mimics key features of inflamed gut mucosa, including DSS‐induced epithelial damage and *C. albicans* tissue invasion. Using this model, we uncover dual protective roles of microbiota‐derived butyrate: (i) stabilization of epithelial adherens junctions and promotion of epithelial renewal, thereby restricting fungal invasion; and (ii) modulation of macrophage function to enhance antifungal activity while attenuating inflammasome‐mediated inflammation. Butyrate pretreatment preserves barrier function, limits fungal translocation, and promotes macrophage viability through the inhibition of histone deacetylase (HDAC) and the suppression of NLRP3 inflammasome activation. These findings position butyrate as a key metabolite in orchestrating epithelial‐immune defense against fungal exacerbation in colitis, supporting its therapeutic and preventive potential in restoring mucosal resilience in IBD.

## Graphical abstract

This study establishes a human immunocompetent colitis‐on‐chip model that mimics inflamed gut mucosa and enables quantitative analysis of *Candida albicans* tissue invasion under DSS‐induced injury. It reveals that microbiota‐derived butyrate simultaneously stabilizes epithelial junctions and reprograms macrophages via HDAC/NLRP3‐dependent mechanisms, thereby limiting fungal penetration, dampening excessive inflammation, and enhancing mucosal resilience in IBD‐like conditions.

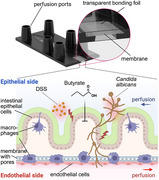

## 1 Introduction

In inflammatory bowel disease (IBD), microbial dysbiosis significantly diminishes butyrate production. Butyrate, an essential short‐chain fatty acid (SCFA) in the intestine, is a key metabolite produced by commensal bacteria during interaction with intestinal epithelial cells (IECs). Butyrate is produced through the fermentation of dietary fibers and plays a critical role in maintaining intestinal homeostasis [1]. This SCFA is the primary energy source for IECs and supports epithelial barrier integrity by upregulating tight junction proteins such as ZO‐1 and E‐cadherin. This reduces epithelial permeability and fosters the gut's ability to prevent pathogen translocation [2, 3].

A reduced abundance of butyrate‐producing bacteria is particularly evident during active disease phases of IBD and is associated with epithelial damage and inflammation [4]. Reduced butyrate levels exacerbate IBD symptoms by compromising epithelial barrier function [5, 6]. In various patient groups, the low abundance of butyrate‐producing bacteria is associated with a permissive environment for the commensal opportunistic fungal pathogen *Candida albicans* to overgrow [7, 8] and increase of its virulence [9]. A mycobiota dysbiosis associated with *C. albicans* overgrowth is a frequently observed phenomenon in IBD patients [6, 10, 11] and has been implicated in further exacerbating inflammation and compromising epithelial barrier integrity [12, 13]. Importantly, an association of *Candida* spp. with the mucosa was found to be linked with disease severity [12, 14].

Restoration of butyrate levels through probiotics or other interventions has been associated with reduced inflammation and improved disease outcomes in IBD [5, 9]. Importantly, butyrate inhibits growth and key fungal virulence factors, including hyphal formation, which is critical for colonization and pathogenesis [15]. This activity is independent of pH effects, suggesting a direct biochemical mechanism of antifungal action [5]. Beyond these direct inhibitory effects on *C. albicans* virulence, butyrate also profoundly impacts host epithelial and immune responses. Understanding how microbiota‐derived butyrate cross‐talks with host cells to contribute to antifungal defense is essential for evaluating the therapeutic potential of butyrate to dampen *C. albicans*‐driven inflammation in IBD settings.

Advanced experimental models can facilitate the dissection of the complex interplay between gut microbes, the host, and metabolites such as butyrate. We, therefore, leveraged intestine‐on‐chip (IoC) platforms to replicate the organotypic microanatomy of the gut, including villus‐ and crypt‐like structures, a functional epithelial barrier, and mucosal immune components [16]. The model was demonstrated to provide physiologically relevant conditions for studying host‐microbe interactions [17]. It incorporates a multi‐layered cellular setup, including intestinal epithelial cells that differentiate into polarized columnar cells under continuous perfusion and endothelial cells that form a vasculature‐like lining. Continuous perfusion facilitates nutrient delivery and maintenance of physiological shear forces, ensuring proper differentiation of intestinal cell subtypes, including absorptive, goblet, and Paneth cells. Innate immune cells, such as macrophages, are integrated into the model, providing an immunocompetent environment. Notably, the absence of physical barriers between microbes and host cells enables detailed analysis of their interactions and the impact of microbes on epithelial integrity and immune responses. Direct apical inoculation of the fungus onto the host epithelial surface, without a separating membrane or gel, facilitates direct quantification of *C. albicans* adhesion, hyphal invasion, and establishment of microcolonies. It also prevents filter‐induced adsorption and enables time‐resolved, unbiased readouts under flow.

Our study focused on butyrate as a potential therapeutic or preventive agent mediating protective effects against *C. albicans* in inflammatory conditions. We developed an advanced immunocompetent colitis‐on‐chip (CooC) model integrating functional monocyte‐derived macrophages. Dextran sodium sulfate (DSS) is the most frequently used chemical agent to induce colitis in experimental animal models of IBD. The compound allows the simulation of acute, chronic, remitting, and recurrent colitis hallmarks by oral administration via drinking water [18]. Similarly, DSS is employed in the CooC model to induce a colitis‐like phenotype that mimics the intestinal inflammation and barrier dysfunction observed in IBD. The combination of our IBD in vitro model with advanced image analysis enabled us to dissect the effects of butyrate in mitigating *C. albicans* infection and inflammation‐driven epithelial damage under reproducibly physiologically relevant conditions.

## 2 Materials and Methods

### 2.1 Ethics Statement

Cell Collection of human Umbilical Venous Endothelial Cells (HUVECs) and Peripheral Blood Mononuclear Cells (PBMCs) Was Approved by the Jena University Hospital Ethics Committee (2021‐2272‐Material, 2018‐1052‐BO), With Written Informed Consent from Donors, in Accordance With the Declaration of Helsinki.

### 2.2 Cell Isolation and Culture

HUVECs were isolated, expanded, and stored at ‐80°C as previously described [19]. Cells were thawed, seeded at 2.5 × 10^4^ cells/cm^2^ in endothelial cell growth medium (EC medium; Promocell, Heidelberg, Germany) supplemented with manufacturer‐provided supplements and 1% v/v Penicillin/Streptomycin (Pen/Strep; Gibco, Thermo Fisher Scientific, Darmstadt, Germany). Medium was refreshed twice weekly, and cells were used up to passage 3.

Human epithelial colorectal adenocarcinoma (Caco‐2) cells were cultured in Dulbecco's Modified Eagle's Medium (DMEM) high glucose (4.5 g/L; Gibco), supplemented with 10% (v/v) fetal calf serum (FCS), 1% (v/v) non‐essential amino acids (NEAA), 1% sodium pyruvate, 0.2% (w/v) gentamycin, and 0.2% holotransferrin (all from Gibco). Medium was exchanged twice weekly, with cells used up to passage 40.

PBMCs were isolated from healthy donors via Ficoll density gradient centrifugation as described previously [20]. Blood collection occurred after informed consent. PBMCs were seeded at 1.0–1.5 × 10^6^ cells/cm^2^ in 6‐well plates with 2 mL X–VIVO 15 medium (Lonza, Cologne, Germany) supplemented with 10% (v/v) autologous human serum, 10 ng/mL granulocyte macrophage colony‐stimulating factor (GM‐CSF), 10 ng/mL macrophage colony‐stimulating factor (M‐CSF) (PeproTech, Hamburg, Germany), and 1% Pen/Strep (Thermo Fisher). Cells were incubated for 1 h at 37°C and 5% CO_2_, washed twice with X‐VIVO medium, and cultured for 24 h before experiments.

### 2.3 Intestine‐on‐Chip Model

BC002 biochips (Dynamic42, Jena, Germany), composed of polybutylene terephthalate (PBT) with a polycarbonate (PC) bonding foil, were used. A polyethylene terephthalate (PET) membrane (12 µm thickness, 8 µm pore size, 1 × 10^5^ pores/cm^2^, cultivation area 1.32 cm^2^) separated the top and bottom chambers. Chips were sterilized with 70% (v/v) ethanol, washed twice with MilliQ water, and coated for 10 min with 0.5 mg/mL collagen‐IV (Sigma, Merck, Darmstadt, Germany). HUVECs (4 × 10^5^ cells) were seeded into the bottom chamber and cultured upside‐down for 48 h at 37°C, 5% CO_2_. Subsequently, 1 × 10^5^ PBMC‐derived monocytes were seeded on HUVECs, and medium was supplemented with 10% (v/v) autologous human serum, 10 ng/mL M‐CSF, and 10 ng/mL GM‐CSF. After 24 h incubation, 5 × 10^5^ Caco‐2 cells were seeded into the upper chamber. Chips were cultured statically for 4 days with daily medium exchange and subsequently connected to circular perfusion (50 µL/min) for 72 h using microfluidic reservoirs and silicon tubing (Dynamic42, Jena, Germany). EC medium perfused endothelial chambers; supplemented DMEM perfused epithelial chambers. The epithelial side was finally stimulated with 100 ng/mL Lipopolysaccharides (LPS; Sigma) for 24 h. Luminal LPS was applied as a physiological MAMP priming cue, enhancing epithelial junctional organization, while avoiding basolateral endotoxemia‐like activation [16]. In parallel, butyrate was included to model microbial metabolite exposure under these conditions.

### 2.4 Colitis‐on‐Chip (CooC) Model

To model colitis, 1.5% (w/v) 40 kDa DSS (MP Biomedicals, Eschwege, Germany) was perfused on the epithelial side for 24–48 h. Sodium‐3‐hydroxybutyrate (butyrate; 1 mm; Sigma) was applied simultaneously with LPS and maintained throughout experiments, with medium exchange every 24 h.

### 2.5 *C. albicans* Cultivation, Growth Assessment, and Infection Model

The well‐established *C. albicans* reference strain SC5314, originally isolated from a patient with disseminated candidiasis [21] was maintained on yeast‐peptone‐dextrose (YPD) agar plates (2% (w/v) agar‐agar, 2% soja‐peptone, 2% glucose; Carl Roth, Karlsruhe, Germany, and 1% (w/v) yeast extract; Serva, Heidelberg, Germany). Stock cultures stored at ‐80°C in YPD medium were streaked onto YPD agar plates and incubated at 30°C for 24 h. These plates served as short‐term stocks for up to three weeks at 4°C. Fresh pre‐infection plates were prepared from short‐term stocks before each experiment and used only once. Before infection, overnight liquid cultures (10 mL YPD, 30°C, 180 rpm, ≤20 h) were centrifuged (10 000 g), washed three times with phosphate‐buffered saline (PBS), and cells counted with a Neubauer chamber. Cells were then resuspended in serum‐free high‐glucose DMEM (4.5 g/L).

To assess the impact of DSS on fungal growth, overnight *C. albicans* cultures were washed with PBS, resuspended in DMEM or EC‐medium ± 1.5% (w/v) DSS, and inoculated into 96‐well plates at 2 × 10^4^ cells/well. Growth at 37°C was monitored by optical density (OD_600_) every 15 min over 24 h using a Tecan Infinite M200 microplate reader (Tecan, Männedorf, Switzerland). Experiments included three independent biological replicates, each with two technical replicates.

Infections with *C. albicans* in the intestine‐on‐chip were conducted as previously described [22]. All infections were performed using continuous perfusion of the model to deliver nutrients and remove catabolites, which are necessary to maintain epithelial tissue integrity. Briefly, the epithelial chamber was infected using 500 µL *C. albicans* suspension (5 × 10^3^ cells/mL) perfused at 25 µL/min for 10 min. During infection, epithelial perfusion was set to linear flow at 25 µL/min, while endothelial perfusion remained circular at 50 µL/min. After 10 min, the residual infection solution was removed, and the reservoirs were washed twice with PBS. Fresh serum‐free DMEM was added, and perfusion continued under linear conditions. Samples for cytokine analysis were collected before infection. For immunofluorescence analysis, infections lasted 12 h. For effluent and fungal burden analyses, infections lasted 24 h, with samples collected after 20 min and at 24 h to assess fungal presence and burden.

To quantify *C. albicans* fungal burden in the CooC model, three compartments were analyzed separately: a) non‐adherent epithelial *C. albicans* present in the waste and supernatant, b) tissue‐invasive fungal cells within the epithelial layer, and c) translocated fungal cells collected from the endothelial chamber. For non‐adherent epithelial cells, waste medium and supernatants were collected from the epithelial side, centrifuged at 10 000 g for 5 min, and resuspended in 1 mL PBS for colony‐forming units (CFU) analysis. Tissue‐invasive fungi were analyzed after lysis of the epithelial compartment using 2% (v/v) Triton X‐100 as described above. Translocated *C. albicans* cells were quantified by collecting all material from the endothelial side, including lysate and supernatant. All samples were serially diluted and plated on YPD agar plates. CFU were counted after incubation for 24 h at 30°C.

### 2.6 Macrophage Live‐Cell Imaging

For macrophage—*C. albicans* cocultures, monocytes were harvested as previously described, seeded in 8‐well coverslips (ibidi, Martinsried, Germany), 24‐, or 96‐well plates according to the experimental setup and differentiated in the presence of 5 ng/mL M‐CSF (R&D Systems, MN, USA) and 5 ng/mL GM‐CSF (R&D Systems, MN, USA) for 5 days. Macrophages were seeded at densities of 6 × 10^4^ (8‐well) or 4 × 10^4^ (96‐well) per plate before infection at a multiplicity of infection (MOI) of 1. Prior to infection, cells were stained for approximately 45 min with 1 µm SYTO deep red dye and 0.5 µm SYTOX green (both Invitrogen, Thermo Fisher, Darmstadt, Germany). For inhibitor treatment, macrophages were pretreated for 24 h with 10 µm suberoylanilide hydroxamic acid (SAHA; Sigma, Merck, Darmstadt, Germany) or 2 h with 100 nm MCC‐950 (Sigma). Plates were transferred to a preheated microscope stage (37°C) for live‐cell imaging. Images of 3–5 randomly selected regions of interest (ROIs) per well were captured every 15–45 min in three channels (transmitted light, SYTOX green, SYTO deep red). Image analysis was conducted using ImageJ, converting fluorescence images into binary formats to quantify total and dead macrophages over time. Cells were also collected post‐infection, diluted, plated on YPD agar, and incubated for 24 h for CFU enumeration.

Determination of fungal burden in macrophage experiments: Macrophages were seeded in 24‐well plates at a density of 1.2 × 10^5^ cells/well and infected with *C. albicans* at an MOI of 1 following pretreatment with 1 mm butyrate or 10 µm SAHA. Supernatants were collected pre‐ and post‐infection for cytokine and lactate dehydrogenase (LDH) assays. Post‐infection, cells were harvested, diluted, and plated onto YPD agar for CFU counting after 24 h incubation.

### 2.7 Effluent Experiments

For effluent analyses after 24‐h infections, the EC medium and DMEM were replaced with phenolred‐free media to facilitate fluorescein isothiocyanate (FITC) Dextran permeability assays. After infection, biochips were disconnected from perfusion, and supernatants were carefully collected from both chambers (200 µL from the epithelial chamber and 150 µL from the endothelial chamber) for subsequent analyses of cytokine levels, LDH activity, and fungal burden. To quantify invasive *C. albicans* colonies remaining within the tissue, cells were lysed by dynamically flushing each chamber with 1 mL of 2% Triton‐X‐100 (Calbiochem, Merck, Darmstadt, Germany). During lysis and flushing, one chamber remained plugged to avoid cross‐contamination. Lysates were subsequently washed once with PBS and analyzed for fungal burden.

### 2.8 FITC Dextran Permeability Assay

For analysis of gut barrier permeability, 4 kDa FITC‐labeled dextran was used at a concentration of 1 mg/mL. To reduce background signal interfering with light measurements, EC‐Medium and DMEM were replaced with phenol‐red‐free media. After 48 h of treatment under different conditions, static FITC‐dextran analysis was performed: Samples for effluent analysis were taken, and the biochip was disconnected from the pump and tubing. 300 µL of phenol‐free DMEM containing 1 mg/mL FITC‐dextran was manually perfused into the epithelial cavity and incubated at 37° and 5% CO_2_ in the dark. After 30 min, samples were taken at first from the epithelial side (200 µL) and then from the endothelial side (150 µL). Smaller sampling volumes were adapted to avoid diluting samples with PBS or phenol‐red‐free medium. To proceed with further fungal burden analysis using CFU, infected samples were centrifuged at 10 000 g for 10 min, and the supernatant was collected for FITC‐Dextran measurement. FITC‐dextran samples were analysed at an excitation wavelength of 490 nm and an emission wavelength of 530 nm using a microplate reader (Tecan Infinite Pro, Tecan, Männedorf, Switzerland). Two standard curves were analyzed at each measurement, starting at 1 mg/mL. FITC‐dextran concentrations were calculated using the standard curve with the best R^2^‐Value. Finally, the gut barrier function was described using the apparent permeability coefficient, calculated with a membrane surface area of 1.32 cm^2^, concentrations of epithelial and endothelial sides, and incubation times of 3600 and 1800 s, respectively [23].

### 2.9 Quantification of Cytokines

For cytokine quantification from endothelial supernatant, the LEGENDplex Multi‐Analyte Flow Assay Kit—Human inflammation Panel 1 (13‐plex) (Biolegend) was used according to the manufacturer's descriptions [24] and measured using a Canto II flow cytometer (Becton Dickinson, Heidelberg, Germany).

### 2.10 LDH Cell Damage Assay

Inflammation‐ and infection‐mediated host cell damage was analyzed by use of the cytosolic enzyme LDH, which is released into the supernatant at cell death. Therefore, the Cytotoxicity Detection Kit (Roche, Mannheim, Germany) was used according to the manufacturer's instructions, and LDH from rabbit muscle (5 mg/mL, Roche) served to generate a standard curve for the determination of LDH activity in supernatants.

### 2.11 Immunofluorescence Staining

At 12 h post‐infection (hpi), biochips were disconnected, and tissues were gently washed three times (epithelial side) and twice (endothelial side) with PBS at room temperature. Tissues were fixed in methanol (Sigma, Merck, Darmstadt, Germany) for 15 min at ‐20°C and rewashed with PBS. Membranes were carefully removed from the chips and sectioned into three equally sized pieces. Blocking and permeabilization were conducted simultaneously for 60 min in PBS containing 3% (v/v) normal donkey serum (Biozol, Eching, Germany) and 0.1% (w/v) saponin (Sigma).

Primary antibodies targeting E‐cadherin (rat; Millipore, Merck), ZO‐1 (rabbit/mouse; Thermo Fisher), Ki‐67 (mouse; BD Bioscience), *C. albicans* (rabbit; Acris Antibodies, Origene, Herford, Germany), VE‐cadherin (goat; R&D Systems, Abingdon, United Kingdom), and CD68 (mouse; BD Bioscience) were applied and incubated either for 90 min at room temperature or overnight at 4°C. Following three washes with PBS containing 0.1% (w/v) saponin, membranes were incubated for 30 min at room temperature with secondary antibodies: donkey anti‐rat‐Cy3, donkey anti‐goat‐Cy3, donkey anti‐mouse‐AF488, donkey anti‐rabbit‐AF488 (all Jackson Research, Biozol, Hamburg, Germany), donkey anti‐rabbit‐AF647 (Invitrogen, Thermo Fisher), and DAPI (Invitrogen, Thermo Fisher). After incubation, membranes were washed twice more with PBS containing 0.1% (w/v) saponin and a final wash with PBS alone. Stained membranes were then mounted onto clean glass slides using fluorescence mounting medium (Dako, Agilent, Waldbronn, Germany). Non‐infected control tissues were processed identically.

### 2.12 Image Acquisition and Preprocessing

#### 2.12.1 Structured Illumination Microscopy

To characterize the structural and functional properties of the host tissue, high‐resolution three‐dimensional images were acquired from both epithelial and endothelial compartments. Multichannel imaging data of the epithelial compartment included E‐cadherin, the tight junction protein ZO‐1, and nuclear staining to visualize epithelial barrier integrity, along with the proliferation marker Ki‐67 to assess epithelial proliferative activity. Multichannel imaging data of the endothelial compartment included VE‐cadherin and nuclear staining to evaluate endothelial junction integrity. Imaging was performed using an AxioObserver Z1 fluorescence microscope equipped with an Apotome and a 20x NA0.8 air objective lens (Carl Zeiss AG, Jena, Germany), which provided sufficient resolution to visualize fine junctional structures. Each field of view covered an area of 447.63 × 335.40 µm^2^, with a voxel size of 0.3225 × 0.3225 × 1 µm in the x, y, and z dimensions, respectively. For assessment of proliferative activity, Ki‐67 and nuclei were imaged using the same setup but with a higher axial resolution, yielding a voxel size of 0.3225 × 0.3225 × 0.75 µm.

In addition, to quantify macrophage presence and activation, images of CD68‐labeled macrophages on the endothelial side were acquired using the same microscope system but with a 10× NA0.4 objective lens. This configuration allowed for imaging of a larger field of view of 895.26 × 670.80 µm^2^ at a voxel size of 0.645 × 0.645 × 1 µm, which was sufficient for capturing broader macrophage distribution patterns without the need for subcellular details.

For each sample, four to six randomly selected, non‐overlapping image stacks were recorded. Optical sectioning of Apotome‐acquired images was performed using the ZEN software (Carl Zeiss AG, Jena, Germany).

#### 2.12.2 Spinning Disk Confocal Microscopy

Confocal microscopy was used exclusively on infected chips to provide a large field of view for visualizing fungal microcolonies and to assess fungal colonization and host–pathogen interactions at 12 hpi.

Multi‐channel three‐dimensional images were acquired using a Zeiss Axio Observer Z1 spinning disk confocal microscope (Carl Zeiss AG, Jena, Germany) equipped with a 10x NA0.45 air objective lens. The fluorescence channels included nuclei, tight junction protein ZO‐1, and *C. albicans*. One image per sample was acquired using tile scanning acquisition to ensure representative coverage of a large area of the sample. Each image consisted of 25 tiles arranged in a 5 × 5 grid, with each tile covering an area of 878.94 × 662.84 µm^2^ at a voxel size of 0.454 × 0.454 × 1 µm, resulting in a total coverage area of 4043.32 × 3049.06 µm^2^.

The raw images were preprocessed using Huygens Professional software (SVI, Hilversum, Holland). This preprocessing involved deconvolution, utilizing a theoretical point spread function and the classical maximum likelihood estimation algorithm. Additionally, chromatic aberration was corrected to account for variations in the refractive properties of different light wavelengths. To estimate the chromatic aberration in images, multicolor beads with a diameter of 500 nm (Thermo Fisher Scientific, Darmstadt, Germany) were diluted 1:10 with PBS, applied on a glass slide, dried, and mounted in fluorescent mounting medium before imaging. Spinning disk confocal microscopy was employed to acquire 3D images of the beads. The chromatic aberration parameters obtained from four independent images were averaged, and the ZO‐1 and C. albicans channels were then aligned with reference to the nuclei channel. The process of deconvolution and chromatic aberration correction was performed on each individual tile. The processed tiles were then saved as TIFF files, and all tiles were subsequently stitched together using the *Stitching* plugin [25] in Fiji [26].

For both structured illumination microscopy and spinning disk confocal microscopy, the number of z‐layers was adjusted to ensure complete volumetric coverage of the relevant biological structures. This was determined based on the thickness of the tissue and, in the case of infected chips, the vertical extent of fungal microcolony growth.

Excitation/emission wavelengths for fluorescence channels were as follows: AlexaFluor647 (653/668 nm), Cy3 (548/561 nm), AlexaFluor488 (488/509 nm), and DAPI (353/465 nm). In addition to fluorescence imaging, brightfield microscopy was employed in parallel using the microscope's transmitted light setting to visualize the membrane pores.

All image data were saved as multi‐channel z‐stacks with 16‐bit depth in the native Zeiss image format (.CZI) for downstream analysis.

### 2.13 Automated Analysis of Chip Image Data

#### 2.13.1 Quantification of CD68 Expression Level

To assess the expression level of CD68‐labeled macrophages, maximum intensity projections (MIPs) were generated from the z‐stacks. Image analysis was performed using CellProfiler v4.2.6 [27]. MIP images were first smoothed using a median filter with a radius of 5 pixels to reduce background noise, followed by binarization using Otsu thresholding to distinguish foreground signal from background. Objects smaller than 50 pixels or larger than 2000 pixels were detected as artifacts and excluded from further analysis. The mean fluorescence intensity (MFI) was then quantified across all foreground pixels per image, providing a measure of overall CD68 expression.

#### 2.13.2 Quantification of Junctional Integrity: E‐Cadherin, ZO‐1, VE‐Cadherin

Segmentation of the junctional proteins E‐cadherin, ZO‐1, and VE‐cadherin was performed using the “Surfaces” module of IMARIS 10.2.0 (Imaris, Andor Technology Ltd., Belfast.). Image stacks were first smoothed with a surface grain size of 2 pixels, followed by background subtraction, with the diameter of the largest sphere set to 1 µm. Following preprocessing, manual thresholding was applied to identify the foreground signal. Although minor adjustments were made per sample to account for variability, the thresholding criteria were maintained consistently within each biological replicate to ensure comparability. To minimize noise, only surface objects exceeding 100 voxels in volume were retained (Figure S1).

#### 2.13.3 Analysis of Proliferation Marker Ki‐67

To assess cellular proliferation, the Ki‐67 channel was analyzed using the “Surfaces” module in IMARIS, following the same image analysis pipeline as for junctional proteins, with adjustments to parameter values. For the identification of all cells, the DAPI‐stained nuclei channel was processed using the “Spots” module. The estimated XY diameter was set to 6 µm, which proved suitable for capturing nuclei of varying sizes. To minimize false positives, spots were filtered using the “Quality” metric to exclude background noise. Subsequently, detected nuclei were classified into two groups based on their shortest distance to the segmented Ki‐67 surfaces. Nuclei located within 1 µm of a Ki‐67 surface were classified as Ki‐67 colocalized. The total number of nuclei was used as a measurement for total cell count, and the percentage of proliferating (Ki‐67^+^) cells was calculated as the ratio of the nuclei colocalized with Ki‐67 to the total number of detected nuclei (Figure S3).

#### 2.13.4 Morphometric Analysis and 3D Localization of C. albicans Microcolonies in Host Tissue

For the quantitative image analysis of confocal‐acquired tile scans, the four‐channel z‐stacks were imported, annotated, and separated into individual channels, i.e., C. albicans, ZO‐1, nuclei, and brightfield in JIPipe [28]. The analysis workflow was separated into three JIPipe compartments i.e., microcolony morphometrics, tissue architecture, and spatial analysis, to incorporate the previously established image‐based analysis framework for C. albicans infection in an intestine‐on‐chip model into JIPipe [22].

Images were enhanced and binarized to segment the C. albicans microcolonies, using parameters identical to those described in our previously established preprocessing pipeline. To remove background noises, ROIs smaller than 3000 µm^3^ and a mean intensity below 3000 were excluded, followed by morphological filtering of ROIs with compactness > 0.5 using the 3D Suite plugin [29]. Branch reconstruction was then applied, allowing for the detection and correction of breaks in segmented microcolony branches of fungal hyphae caused by locally decreased fluorescence signal, thereby restoring them in the 3D images (Figure S4). Under optimal conditions, fungal microcolonies can undergo excessive growth, leading to contact between adjacent structures. To ensure precise morphometric analysis of individual microcolonies, a watershed‐based split algorithm was implemented to accurately differentiate and separate overlapping microcolonies (Figure S5). For the microcolony image shown in Figure 6a) surface reconstruction was done using IMARIS 10.0.1.

Additionally, a pixel‐based heatmap of epithelial tissue thickness was generated using the nuclei and brightfield channels, applying the same parameters as the previously established approach. Thickness distributions were obtained by sampling 10 000 randomly selected points per image, yielding representative thickness profiles for each condition, depicted in one violin plot for each condition. Due to the large sample size, effect sizes were calculated using Hedges' g (effect size of g = 0.2 signifies a small effect, of g = 0.5 medium effect, and of g≥0.8 large effect. To ensure accuracy, regions corresponding to chip edges and areas of tissue damage were excluded from the final map (Figure S6).

Tissue penetration by fungal microcolonies (TP) was quantified using a Dice coefficient formula:

(1) $$TP=2\times \;\frac{\sum_{i=1}^{N}{V_{i}}_{epithelial}\cap V_{tissue}}{V_{tissue}+\sum_{i=1}^{N}{V_{i}}_{epithelial}}$$

where *V_i_*_*epithelial*_ is the volume of the i^th^ fungal microcolony located within the epithelial compartment, *V_tissue_* is the total epithelial tissue volume, *V_i_*_*epithelial*_∩*V_tissue_* ​ represents the volume of i^th^ fungal microcolony within the epithelial tissue, and N is the total number of microcolonies. Incorporating tissue volume into the calculation is essential because epithelial thickness can vary across conditions. By accounting for the tissue volume, the metric adjusts for scenarios with varied tissue amount, ensuring accurate representation of fungal penetration relative to the tissue size.

Similar to our previous study [22], vascular invasion (VI) was calculated as follows:

(2) $$VI\;=\frac{\sum_{i=1}^{N}{V_{i}}_{vascular}}{\sum_{i=1}^{N}V_{i}}\;$$

where *V_i_*_*vascular*_ is the volume located within the vascular compartment, and *V_i_* is the total volume of the i^th^ fungal microcolony, respectively. This formula quantifies the extent of vascular invasion by calculating the ratio of the fungal microcolony volume that has invaded the vascular area relative to the total fungal volume.

Moreover, analysis of epithelial tissue thickness, beyond its integration into the fungal microcolony analysis pipeline, was applied broadly across Apotome‐acquired images.

#### 2.13.5 Comparison of Static and Perfused Culture Conditions

To Assess the Effect of Continuous Perfusion on Cell Function and Tissue Organisation in the Intestine‐on‐chip Model, Chips Were Assembled and Cultured as Described Above and Assigned to One of Two Culture Regimes. “Flow” Chips Were Continuously Perfused on both Sides at 50 µL/Min throughout the Culture Period. “Static” Chips Received Only Brief Daily Perfusion at 50 µL/Min for 30 Min/Day. After 72 h of Culture, 100 Ng/Ml LPS Was Introduced to the Epithelial Compartment in both Conditions. After a Further 48 h of Incubation, Corresponding to the Timepoint at Which Infection Experiments Are Initiated in the Main Study, Chips Were Processed for either Bulk RNA Sequencing or Immunofluorescence Analysis.

For Immunofluorescence Analysis, Membranes Were Extracted and Divided. Epithelial Membranes Were Fixed With Ice‐cold 100% Methanol for 10 Min, and Endothelial Membranes With 4% PFA for 10 Min at Room Temperature. Epithelial Sections Were Stained With Anti‐ZO‐1 (goat, 1:100, Invitrogen, PA5‐19090), and Anti‐HLA‐DR/DQ/DP (rabbit, 1:100, Bio Techne, NBP2‐79709). Endothelial Sections Were Stained With CD31‐FITC (mouse, 1:50, BD, 555445) and Anti‐HLA‐DR/DQ/DP. Secondary Antibodies Included Donkey Anti‐goat AF647 (1:200, Invitrogen, A21447), Donkey Anti‐rabbit Cy3 (1:200, Jackson ImmunoResearch, 711‐165‐152), and Donkey Anti‐rabbit AF647 (1:200, Invitrogen, A31573). Nuclei Were Counterstained With DAPI (1:1000, Invitrogen, D3571). CD31‐FITC Was Applied After Secondary Antibody Incubation. Multicolour Z‐stack Images Were Acquired and Processed Into Maximum Intensity Projections as Described Above. Nuclei Counts, Macrophage Counts (HLA‐DR/DQ/DP‐positive regions of interest), CD31 Network Mean Fluorescence Intensity, and ZO‐1 Network Mean Fluorescence Intensity Were Quantified Using JIPipe.

#### 2.13.6 Bulk RNA Sequencing and Transcriptomic Analysis

For RNA Isolation, Chips Were Washed With 1 Ml PBS +/+ per Channel. The Upper Bonding Foil Was Removed, and the Tissue‐covered PET Membrane Was Extracted and Transferred Into 350 µL RLT Buffer (Qiagen) at Room Temperature for 5 Min. Samples Were Vortexed, the PET Membrane Removed, and RNA Isolation Was Performed Using the RNeasy Kit (Qiagen) According to the Manufacturer's Protocol. Directional mRNA Library Preparation via Poly(A) Enrichment and Strand‐specific Sequencing Was Performed on the NovaSeq X Plus Series (PE150) by Novogene GmbH (Munich, Germany) With a Targeted Sequencing Depth of 20 Million Reads per Sample.

Raw Paired‐end FASTQ Files Were Quality‐checked Using FastQC and Aligned to the human Reference Genome GRCh38 Using STAR. Gene‐level Read Counts Were Obtained With featureCounts Using the Ensembl Gene Annotation. Genes With Low Expression Were Filtered by Retaining Genes With at Least 10 Counts in at Least 3 Samples. Differential Gene Expression Analysis Was Performed Using PyDESeq2 With Condition as the Design Factor and Static Samples as the Reference Level. Genes With an Adjusted p‐value < 0.05 and an Absolute log2 Fold Change > 1 Were Considered Differentially Expressed. Principal Component Analysis (PCA) Was Performed on log2‐transformed Counts per Million (log2‐CPM) Values to Assess Sample Clustering. Functional Enrichment Analysis Was Performed With GSEApy Using Ranked Differential Expression Results and Gene Sets From the Gene Ontology Biological Process Database. Macrophage‐associated Transcriptional Changes Were Assessed Using Curated Marker Panels Representing Macrophage Identity (CD14, ITGAM, CSF1R, AIF1, HLA‐DRA), Pro‐inflammatory (IL1B, TNF, CXCL8, STAT1), Anti‐inflammatory (CD163, MRC1, MERTK, TGFB1, PPARG, STAT6, CHI3L1), and Phagocytic/Complement‐associated Genes (C1QA, C1QB, C1QC, MSR1, TYROBP, FCER1G, LST1, CTSB, CTSD).

### 2.14 Statistical Analyses

Statistical analyses were performed using GraphPad Prism v8.3.0 and *scikit‐posthocs* v0.10.0 library in *Python* v3.10. For each dataset, the choice of statistical test depended on both the experimental design and the sample size per condition. For datasets with ≥10 observations per condition, the normality of each sample distribution was assessed using Shapiro‐Wilk tests in combination with visual inspection of quantile‐quantile plots. When data were normally distributed, parametric tests such as unpaired t‐tests, one‐way and two‐way ANOVA with multiple comparisons were applied. If normality assumptions were not met, nonparametric alternatives were used instead, specifically the Kruskal–Wallis test followed by Benjamini‐Hochberg‐corrected Dunn post hoc comparisons. In contrast, datasets with <10 observations per condition were analyzed using parametric methods only, as non‐parametric tests provide limited statistical power with extremely small sample sizes. A *p*‐value < 0.05 was considered statistically significant. Exact p‐values are reported for p < 0.07, while p < 0.0001 is represented by “^****^” in the figure legends. In cases involving large sample sizes (e.g., in the thousands), effect sizes were calculated using Hedges’ g, where a value of g = 0.2 is considered a small effect, g = 0.5 represents a medium effect, and g ≥ 0.8 indicates a large effect. Exact g values for g < 0.4 are reported in the figure legends.

### 2.15 Experimental Design and Condition Nomenclature

To improve readability, we use standardised labels for all experimental arms. Each label specifies DSS status + duration, butyrate (BUT) schedule, and (where applicable) infection and macrophage addition: No DSS 24 h/48h: vehicle control under flow for 24 h or 48 h (no DSS); + DSS 24 h/48h: 1.5% DSS apical for 24 or 48 h under flow; DSS only: DSS without butyrate (duration indicated by “24 h” or “48 h”); BUT only: 1 mm butyrate apical under flow (no DSS; duration matched to the paired DSS cohort); BUT + DSS (pre‐exposure): butyrate started 24 h before DSS and was maintained during DSS exposure; BUT + DSS (co‐exposure): butyrate added together with DSS and maintained thereafter (used only where explicitly indicated); + *C. albicans*: addition of *C. albicans* at the infection timepoint defined in “Infection model” (MOI and time window as specified there); absence denotes non‐infected; ± MP: presence or absence of macrophages in the chip model; Pre: Pre‐treatment of the tissue with the respective compound 24 h before the tissue was infected with *C. albicans*; Co‐T: Co‐treatment of the tissue with the respective compound parallel to infection with *C. albicans* for indicated times

## 3 Results

### 3.1 Establishment of CooC Model

**FIGURE 1:**
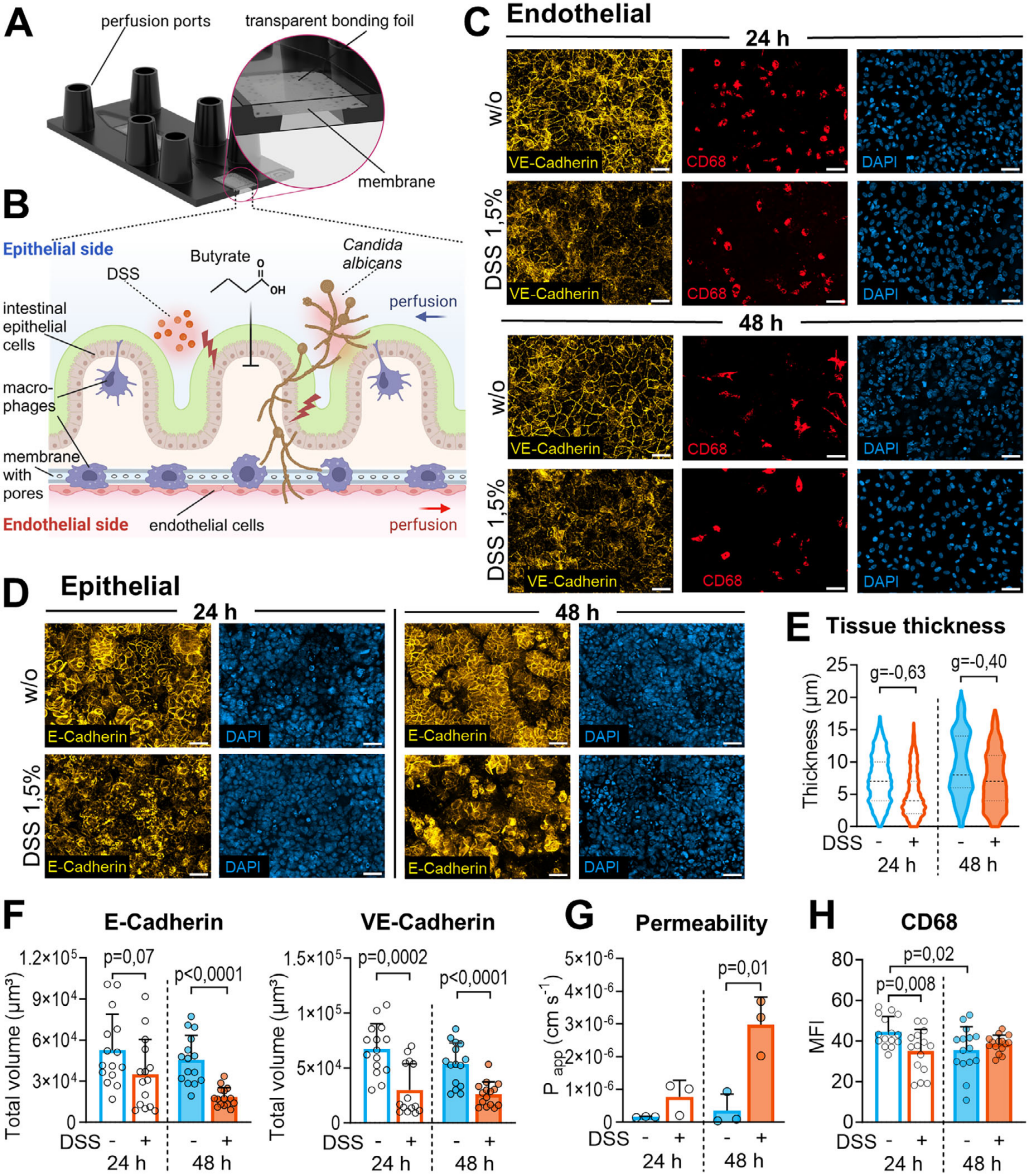
Design and functional characterization of a human CooC model. (A) The biochip holds two cavities on the chip, each with a suspended porous membrane that separates two chambers and serves as a cell substrate. A transparent bonding foil allows visual inspection and imaging of the tissue. Access to the chambers is provided via two perfusion ports. (B) Schematic representation of the CooC model. Intestinal epithelial cells (Caco‐2, brown) self‐organize under flow into a three‐dimensional intestinal tissue with villi‐ and crypt‐like structures [16]. The porous membrane separates it from endothelial cells (HUVEC, red), forming an endothelial lining. Monocyte‐derived macrophages (dark violet) in the model represent innate immune system cells. DSS and butyrate treatments and infection with C. albicans are performed at the epithelial side of the model. Illustrations were created using biorender.com. (C) Immunofluorescence MIP images of endothelial tissue after 24 and 48 h treatment with DSS 1,5% or without treatment (w/o) stained for VE‐cadherin (dark yellow), CD68 (macrophages, red), and DAPI (nuclei, blue). (D) Immunofluorescence MIP images of epithelial tissue after 24 and 48 h treatment with DSS 1,5% or without treatment (w/o). The tissue was stained for E‐cadherin (dark yellow) and DAPI (nuclei, blue). Representative images for endothelial and epithelial cell layers from three independent experiments. Scale bar, 50 µm. (E) Thickness of the intestinal epithelium in µm after 24 and 48 h with (+, orange) or without DSS treatment (‐, blue). Statistical analysis was performed using Hedges'g due to the large sample size. (F) Quantitative Immunofluorescence data shown in bar graphs, as total volume (µm^3^) per image for E‐cadherin network and VE‐cadherin network, after 24 and 48 h with (+, orange) or without DSS treatment (‐, blue). (G) Apparent Permeability of the intestinal barrier after 24 and 48 h with (+, orange) or without DSS treatment (‐, blue). (H) MFI per image for CD68^+^ macrophages, after 24 and 48 h with (+, orange) or without DSS treatment (‐, blue). (E–H) Data from three independent experiments is shown. (F–H) Bars express mean values ± SD, with single values shown as circles. Statistical testing was performed using a two‐tailed *t*‐test for each time point. Only *p*‐values ≤0,07 are shown.

To investigate the effects of inflammation and therapeutic modulation in a human‐relevant setting, we employed an immunocompetent intestine‐on‐chip model that recapitulates key structural and cellular features of the intestinal barrier (Figure 1A). The model consists of polarized epithelial and endothelial cell layers separated by a porous membrane, co‐cultured with monocyte‐derived macrophages under continuous perfusion to mimic physiological shear stress [16].

To validate that continuous perfusion is essential for establishing a functional tissue environment in the intestine‐on‐chip model, we compared chips cultured under continuous flow (50 µL/min) with chips receiving only brief daily perfusion (30 min/day) over 5 days. Both conditions received 100 ng/ml LPS in the epithelial compartment after 72 h to match the priming conditions used in the main experiments. Immunofluorescence analysis revealed that continuous perfusion promoted a well‐organised CD31‐positive endothelial junctional network, whereas static cultures showed fragmented, poorly organised CD31 staining (Figure S12A). On the epithelial side, continuous perfusion resulted in the formation of a dense ZO‐1‐positive tight junction network characteristic of a differentiated intestinal epithelium, while static cultures showed sparse, discontinuous ZO‐1 expression (Figure S12B). HLA‐DR/DQ/DP‐positive cells, indicative of macrophage presence and antigen‐presenting capacity, were detectable in both the endothelial and epithelial compartments under continuous flow but were sparse or absent under brief perfusion (Figure S12C,D). In this system, HLA‐DR/DQ/DP serve as specific markers for macrophages and antigen‐presenting cells.

To further characterize the molecular basis of these differences, we performed bulk RNA sequencing (n = 3 biological replicates per condition). Principal component analysis demonstrated clear separation of briefly and continuously perfused samples along PC1 (Figure S13A). Differential gene expression analysis identified 2192 significantly upregulated and 1966 significantly downregulated genes under continuous flow (adjusted *p* < 0.05, |log2FC| > 1), demonstrating that continuous perfusion fundamentally reshapes the transcriptomic landscape of the co‐culture model (Figure S13B). Gene set enrichment analysis revealed distinct functional programs associated with continuous flow (Figure S13C). Pathways related to immune activation were significantly enriched, including MHC class II protein complex assembly, peptide antigen assembly with MHC class II, regulation of T cell activation, and leukocyte adhesion to vascular endothelial cells. Concurrently, MHC class I antigen‐processing pathways were downregulated, indicating a shift from endogenous peptide presentation toward professional exogenous antigen presentation, characteristic of mature antigen‐presenting cells. Continuous flow further promoted enrichment of fatty acid metabolic pathways, including fatty acid beta‐oxidation and long‐chain fatty acid metabolism, as well as mitochondrial gene expression and tRNA processing programs, consistent with increased metabolic and translational activity under perfusion.

To specifically assess the effect of perfusion on macrophage maturation, we examined curated gene panels associated with macrophages (Figure S13D). Continuous flow promoted the coordinated upregulation of macrophage identity markers (CD14, ITGAM, CSF1R, AIF1, HLA‐DRA), pro‐inflammatory mediators (IL1B, TNF, CXCL8, STAT1), anti‐inflammatory regulators (CD163, MRC1, MERTK, TGFB1, PPARG, STAT6, CHI3L1), and phagocytic/complement components (C1QA, C1QB, C1QC, MSR1, TYROBP, FCER1G, LST1, CTSB, CTSD). The balanced co‐upregulation of both pro‐inflammatory and anti‐inflammatory programs indicates that perfusion promotes the maturation of monocyte‐derived macrophages toward a tissue‐resident phenotype capable of immune surveillance while maintaining inflammatory homeostasis. These results establish that continuous perfusion is essential for generating a physiologically relevant, immunocompetent intestinal tissue model. All subsequent experiments were therefore performed under continuous flow conditions.

In this model, a colitis‐like phenotype was induced by perfusing the epithelial side with DSS for up to 48 h, while the treatment effects of butyrate were tested by pre‐conditioning the epithelial compartment with 1 mM butyrate starting 24 h before DSS exposure (Figure 1B). Treatment with 1.5% DSS for up to 48 h induced a colitis‐like phenotype in the immunocompetent intestine‐on‐chip model in a time‐ and dose‐dependent manner (Figure 1C–H; Figure S2). While in non‐treated tissue, a well‐differentiated and intact adherens junction complex protein (AJCP) network was observed, as shown by immunofluorescence microscopy, the DSS colitis phenotype was characterized by the intestinal barrier breakdown. DSS treatment for 24 and 48 h led to pronounced rupture of VE‐cadherin in the endothelial layer (Figure 1C; Figure S2B) and E‐cadherin in the epithelial layer (Figure 1D; Figure S2A). Quantitative analysis revealed that DSS significantly reduced the total 3D junctional volume of both junctional proteins (Figure 1F), indicating progressive disintegration of the AJCP network. This structural disruption corresponded with a marked loss of intestinal barrier function, as measured by increased permeability to 40 kDa FITC‐dextran after 48 h of DSS exposure (Figure 1G). Additionally, epithelial tissue thickness was significantly reduced following DSS treatment (Figure 1E). DAPI staining revealed that the overall abundance of epithelial and endothelial nuclei remained unchanged (Figure 1C,D), suggesting that barrier breakdown was not due to widespread cell loss. However, the expression level of CD68‐positive monocyte‐derived macrophages was downregulated significantly after 24 h DSS treatment (Figure 1H).

**FIGURE 2:**
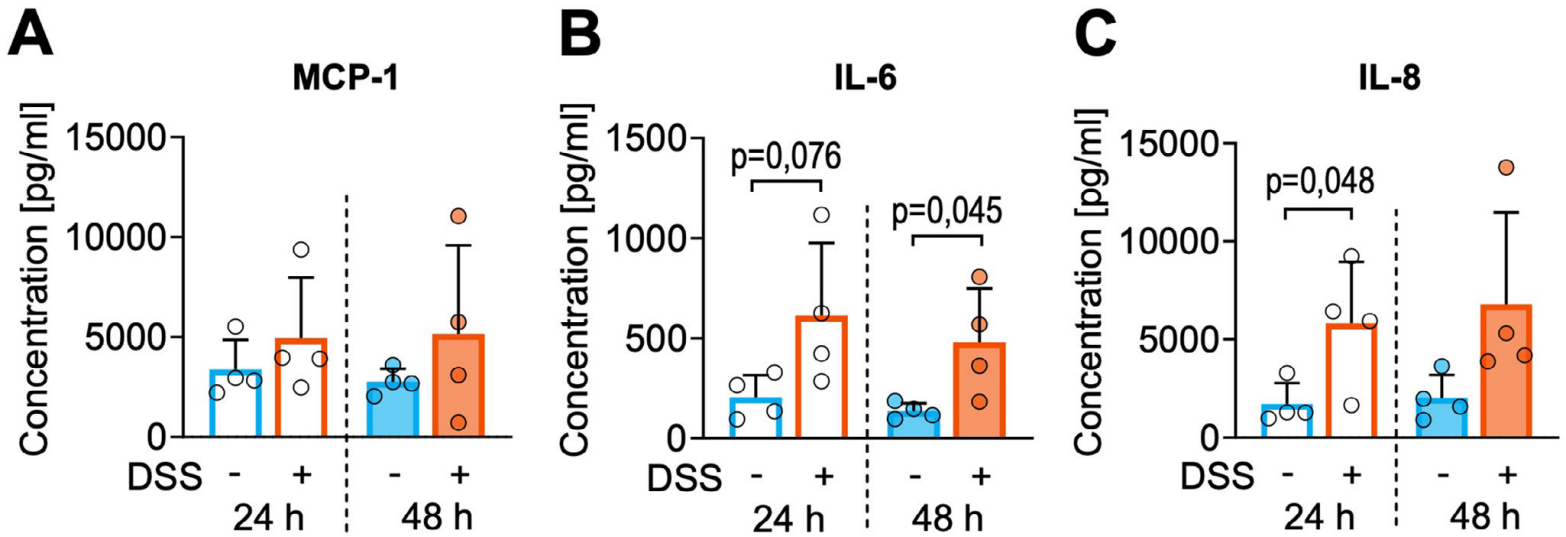
Cytokine release in the CooC model. Quantification of (A) MCP‐1, (B) IL‐6, and (C) IL‐8 levels (pg/mL) in endothelial effluents after 24 and 48 h of culture, with (orange) or without (blue) DSS treatment. Bars represent mean values ± SD; individual data points are shown as circles. Statistical analysis was performed using two‐tailed *t*‐tests for each time point.

We quantified cytokine release into the endothelial compartment to assess the degree of inflammation. DSS treatment triggered a pro‐inflammatory immune response detectable as early as 24 h and persisting through 48 h (Figure 2A–C). Levels of monocyte chemoattractant protein‐1 (MCP‐1), interleukin‐6 (IL‐6), and IL‐8 were elevated, consistent with observations from clinical and in vivo studies (Table 1) [30, 31, 32, 33, 34].

**TABLE 1:** Endothelial cytokine profiles. Absolute concentrations of 12 cytokines (pg/mL) at 24 h (left) and 48 h (right), with mean ± SD indicated in brackets. Statistical analysis was performed using two‐way ANOVA with Tukey's multiple comparisons test.

	24 h (two‐way ANOVA p = 0.01)		48 h (two‐way ANOVA p = 0.03)	
	w/o	+ DSS	w/o	+DSS
IFN‐γ	5.0 ± 2.6	6.3 ± 0	5.1 ± 2.3	6.8 ± 1.0
IL‐1β	13.2 ± 4.7	15,7 ± 0.3	15.6 ± 0	15.6 ± 0
TNFɑ	8.6 ± 4.5	12.3 ± 4,2	8.3 ± 5.0	12.7 ± 3.6
MCP‐1	3386 ± 1464	4940 ± 3040	2775 ± 641.4	5158 ± 4434
IL‐6	206.2 ± 109	612.9 ± 364,0	136.5 ± 39,6	480.8 ± 269.0
IL‐8	1713 ± 1063	5819 ± 3132	2031 ± 1164	6790 ± 4692
IL‐10	5.2 ± 3.9	6.4 ± 4.7	4.8 ± 2.8	4.3 ± 0.9
IL‐12p70	5.2 ± 6.0	5.7 ± 4.0	4.9 ± 5.2	3.9 ± 2.0
IL‐17A	2.0 ± 2.3	3.2 ± 4.1	1.9 ± 1.3	1.8 ± 1.2
IL‐18	39.3 ± 26.7	64.8 ± 54.5	39.3 ± 16.1	46.7 ± 27.1
IL‐23	9.6 ± 11.0	14.8 ± 11.5	9.3 ± 11.8	11.1 ± 6.2
IL‐33	32.7 ± 31.2	34.5 ± 20.7	28.5 ± 24.2	25.8 ± 7.6

### 3.2 Butyrate Ameliorates DSS‐Induced Tissue Damage

**FIGURE 3:**
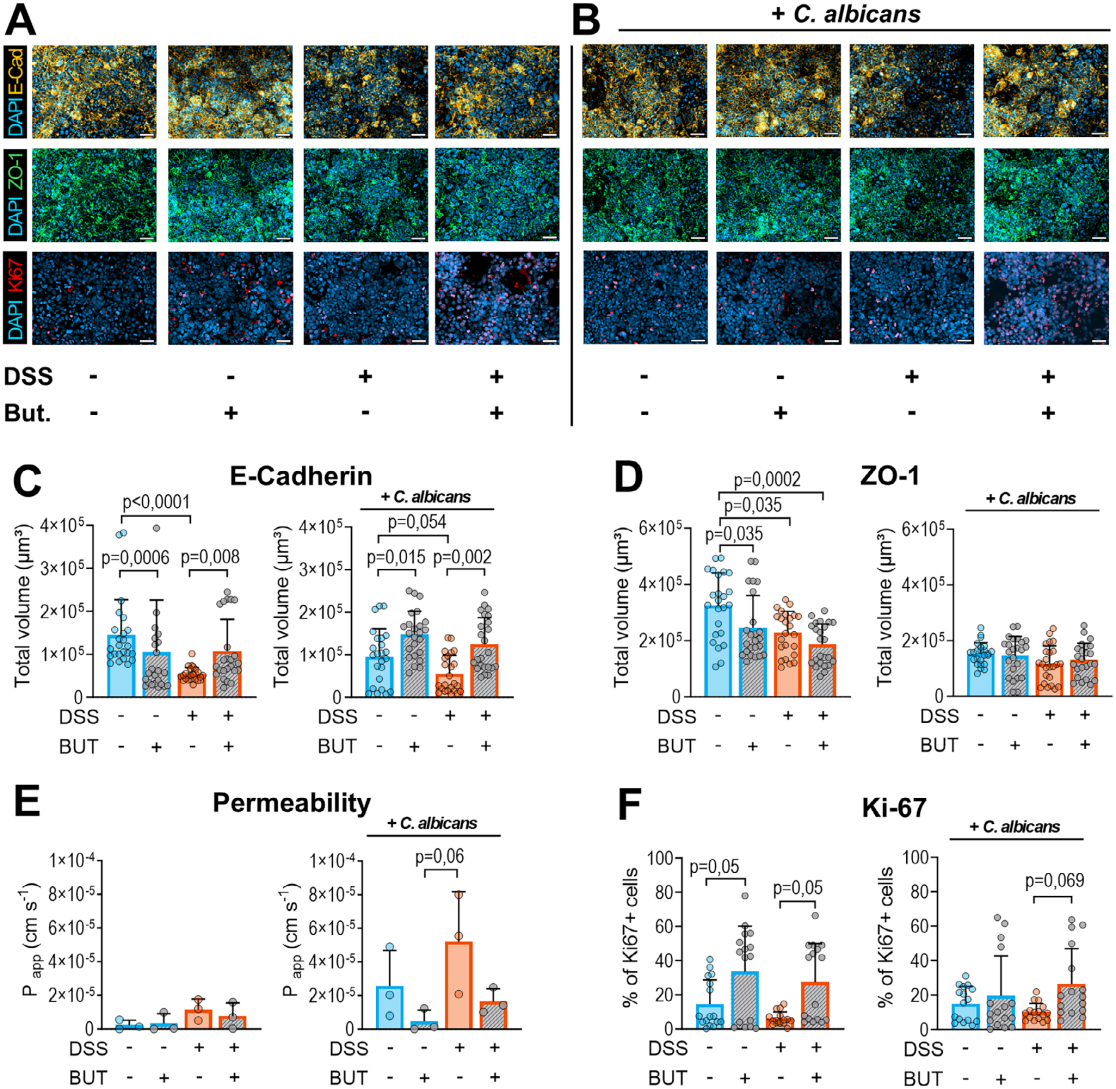
Effects of preventive butyrate treatment on epithelial host tissue in the CooC model. (A, B) Representative immunofluorescence MIP images of epithelial tissue upon 36 h DSS and/or butyrate treatment from four independent experiments under (A) non‐infected and (B) infected conditions. The tissue was stained for E‐cadherin (dark yellow), ZO‐1 (green), Ki‐67 (red), and DAPI (nuclei, blue). Representative images from four independent experiments. Scale bars, 50 µm. (C) E‐cadherin network, (D) ZO‐1 network, (E) apparent intestinal barrier permeability, and (F) percentage of Ki‐67 positive cells within the epithelial tissue per image. Data from three‐dimensional deconvolutions of immunofluorescence z‐stacks. (C, D, F) Quantitative IF data shown in bar graphs as the total volume per image (in µm^3^), bars express mean values ± SD of non‐treated (blue) and DSS‐treated tissue (orange), and after the treatment with butyrate (green), under non‐infected (left) and infected conditions (right), with single values shown as circles. Statistical testing for (C, D, F) was performed using a Kruskal–Wallis test followed by Benjamini–Hochberg corrected Dunn post‐hoc comparisons test. Data from four independent experiments are shown. Statistical testing for (E) was performed using one‐way ANOVA with Tukey's multiple comparisons test. Data from three independent experiments are shown.

Next, we assessed the therapeutic effects of butyrate in the CooC model. As a crucial pathobiont associated with IBD severity, we introduced *C. albicans*. A concentration of 1 mm butyrate was added to the epithelial perfusion medium 24 h prior to DSS treatment. This pre‐treatment preserved the AJCP network, which was otherwise disrupted by DSS (Figure 3A,B). To analyze epithelial integrity in more detail, three‐dimensional quantification of deconvolved z‐stack immunofluorescence images was performed. DSS significantly reduced the overall E‐cadherin network volume, while butyrate treatment preserved it in both non‐infected and *C. albicans*‐infected models (Figure 3C). E‐cadherin network volume represents the 3D‐integrated volume of E–cadherin–positive adherens junctions throughout the epithelium. Its reduction indicates junctional fragmentation and lateral membrane shortening, which is consistent with barrier impairment caused by DSS. In contrast, ZO‐1 did not exhibit a comparable response. Its network volume was diminished upon *C. albicans* infection and not preserved by butyrate treatment (Figure 3D). Nonetheless, under infected conditions, butyrate showed a trend toward preventing epithelial barrier permeability (Figure 3E). Moreover, butyrate enhanced epithelial regeneration, as indicated by increased Ki‐67 expression. In non‐infected tissue, butyrate promoted epithelial proliferation regardless of DSS exposure. Under infection, overall Ki‐67 expression decreased, but showing a tendency for butyrate to increase Ki‐67 levels in the DSS‐treated colitis model (Figure 3F). DSS treatment further reduced tissue thickness under non‐infected conditions and upon infection with *C. albicans*, an effect that was partially reversed by butyrate in the presence of infection (Figure S7).

**FIGURE 4:**
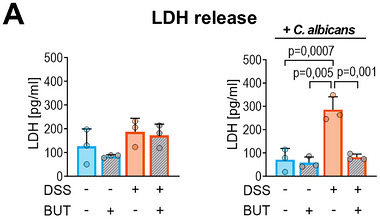
Endothelial effluent readouts of butyrate treatment in the CooC model. (A) Quantification of LDH release from the endothelial side after 48 h. Bars show mean values ± SD for non‐treated (blue), DSS‐treated (orange), and butyrate‐treated (hatched bars) tissues, under non‐infected (left) and infected (right) conditions. Individual data points are shown as circles. Statistical analysis was performed using one‐way ANOVA with Tukey's multiple comparisons test; only *p*‐values ≤ 0.07 are reported. (Data from three independent experiments are shown).

At the endothelial side of the colitis model, the VE‐cadherin network and macrophages expressing CD68, a pan‐macrophage marker, were analyzed by two‐dimensional analysis of z‐stack‐acquired immunofluorescence images. In non‐infected tissue, the total junctional VE‐cadherin volume did not reveal significant changes. In contrast, under infected conditions, the total of VE‐cadherin network volume was reduced by DSS treatment, resulting in a higher level of network fragmentation (Figure S8A–C). Here, butyrate treatment significantly restored VE‐cadherin expression in the infected DSS‐induced colitis model, reducing VE‐cadherin network fragmentation. The expression level of CD68 on monocyte‐derived macrophages showed a slight increase induced by butyrate treatment in the presence of DSS (Figure S8D). In addition, supernatants of the endothelial tissue were analyzed. Here, a significantly elevated level of cell death was observed under infected and DSS‐treated conditions, which was quantified by the release of LDH. This increase was prevented by butyrate treatment (Figure 4A). Furthermore, the overall relative release of pro‐inflammatory cytokines increased by DSS treatment was decreased by butyrate treatment, under non‐infected, as well as under infected conditions (Tables 2 and 3; Figure S9).

**TABLE 2:** Absolute concentrations (pg/mL) of 12 cytokines released from the endothelial compartment under non‐infected and (B) *C. albicans*‐infected conditions, following treatment with DSS (1.5%) and/or butyrate (1 mm). Values represent mean ± SD from four independent experiments. Cytokine changes with statistically significant differences are indicated by the addition of p‐value (*p* ≤ 0.05, two‐way ANOVA with Tukey's test). (data from three independent experiments are shown).

	w/o	+ BUT	+ DSS	+ DSS/BUT
IFN‐γ	6.2 (±5.4)	6.6 (±7.2)	5.8 (±2.1)	5.7 (±3.1)
IL‐1β	13.0 (±12.4)	74.9 (±91.7)	22.2 (±8.1)	8.0 (±6.0)
TNFɑ	7.5 (±5.9)	7.5 (±4.7)	15.6 (±5.4)	7.2 (±2.8)
MCP‐1	2082 (±1699)	3323 (± 2375)	1875 (± 1451)	1486 (±1290)
IL‐6	106 (±92)	111 (±24.0)	164 (±174)	82.8 (±51.0)
IL‐8	1802 (± 2174)	1447 (± 706) vs. w/o p = 0,0007	5525 (± 713) vs. + BUT p = 0,002	2797 (±2425) vs. + DSS p = 0,04
IL‐10	4.4 (±1.8)	17.8 (±21.2)	4.1 (±1.6)	3.7 (±3.1)
IL‐12p70	5.7 (±14.2)	5.5 (±3.8)	5.0 (±1.6)	4.6 (±3.5)
IL‐17A	1.2 (±0.6)	1.2 (±0.7)	1.3 (±0,9)	1.4 (±0.9)
IL‐18	71.1 (± 39.9	72.4 (±41,9)	84.6 (±37.4)	67.1 (±38.6)
IL‐23	8.2 (±5.9)	7.8 (±2.4)	11.8 (±6.6)	7.2 (±4.5)
IL‐33	179 (±141)	128 (±181)	347.4 (±212)	85.7 (±70.9)

**TABLE 3:** Absolute concentrations (pg/mL) of 12 cytokines released from the endothelial compartment under *C. albicans*‐infected conditions, following treatment with DSS (1.5%) and/or butyrate (1 mm). Values represent mean ± SD from four independent experiments. Cytokine changes with statistically significant differences are indicated by the addition of *p*‐value (*p* ≤ 0.05, two‐way ANOVA with Tukey's test). (data from three independent experiments are shown).

	w/o	+ BUT	+ DSS	+ DSS/BUT
IFN‐γ	20.2 (±23.0)	8.7 (±5.5)	8.0 (±4.9)	3.5 (±0.5)
IL‐1β	41.2 (±36.6)	29.4 (±19.7)	37.2 (±12.2)	20.9 (±14.0)
TNFɑ	17.6 (±8.4)	10.5 (±4.5)	45.4 (±37.5)	14.4 (±7.1)
MCP‐1	9983 (±11937)	3941 (±3020) vs w/o p = 0,005	3274 (±3659)	1198 (± 1001)
IL‐6	1528 (±2561)	88.6 (±65.7)	1567 (±2526)	66.3 (±25.0)
IL‐8	2983 (±2533)	3885 (±3787)	6714 (±4602)	3526 1961
IL‐10	12.0 (±11.4)	7.3 (±1.6)	5.2 (±2.6)	3.0 (±1.4)
IL‐12p70	8.9 (±4.7)	9.4 (±3.0)	8.1 (±4.5)	4.3 (±0.6)
IL‐17A	1.4 (±0.6)	1.2 (±0.8)	1.2 (±0.6)	0.9 (±0.7)
IL‐18	80.6 (±7.2)	92.6 (±36.2)	82.1 (±32.4)	76.6 (±4.3)
IL‐23	13.1 (±10.3)	16.3 (±14.4)	16.0 (±4.3)	8.1 (±5.3)
IL‐33	63.9 (±16.4)	57.7 (±39.7)	58.2 (±18.2)	25.4 (±9.3)

### 3.3 *C. albicans* Infection is Mitigated by Butyrate in the CooC Model

**FIGURE 5:**
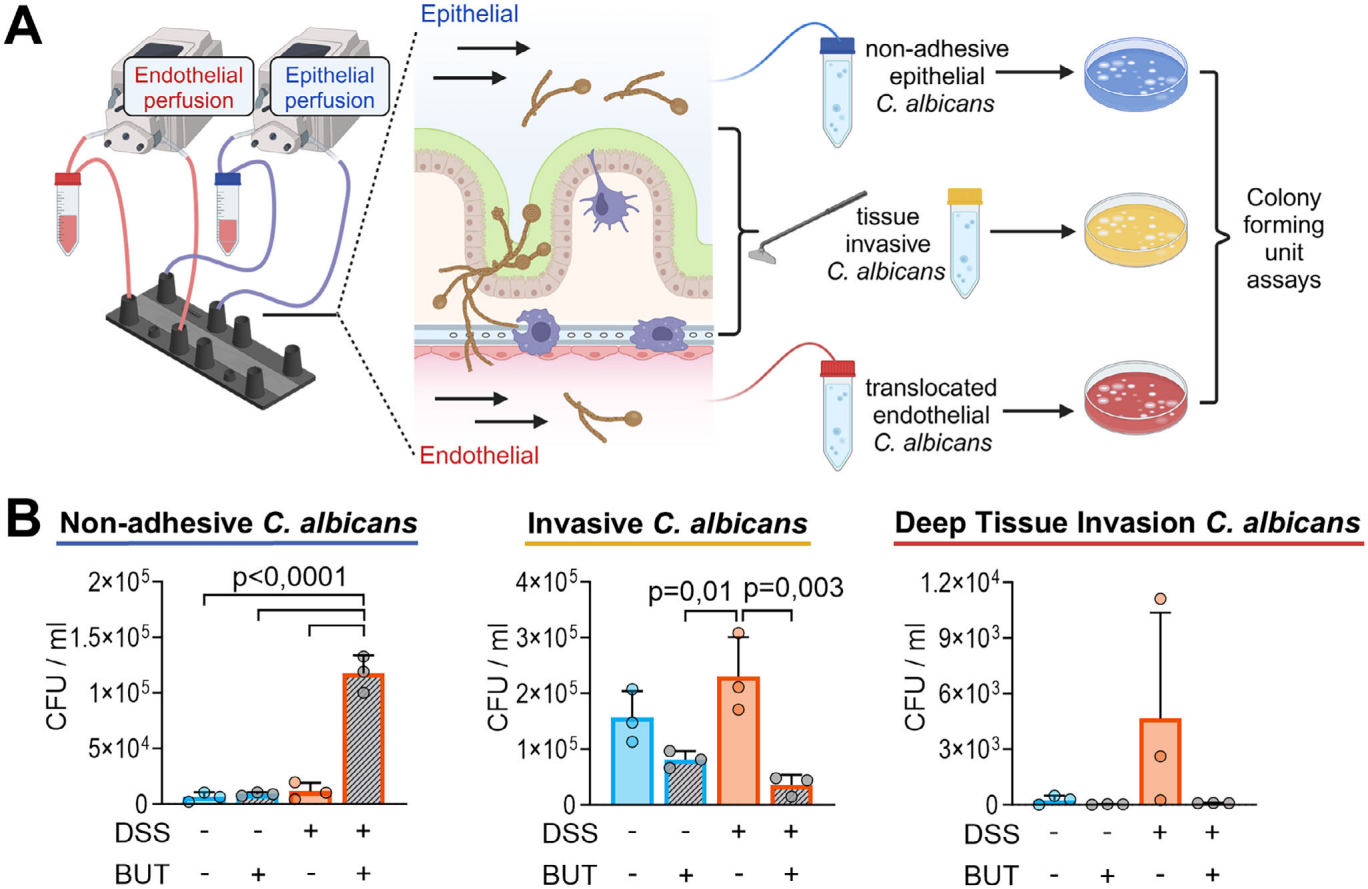
Fungal burden analysis in the CooC model. (A) Schematic illustration of fungal burden analysis in the CooC model. CFU in three different compartments were analyzed separately. Non‐adhesive epithelial C. albicans cells within the epithelial washout that detached easily (blue), tissue invasive C. albicans hyphae that were growing invasively within the epithelial tissue (yellow), and translocated C. albicans that translocated over the membrane to the endothelial side and further disseminated within the endothelial chamber (red). Illustrations were created using biorender.com. (B) Fungal burden results, quantified as CFU/ml 24 hpi with C. albicans, with (+, orange) or without DSS treatment (‐, blue) and upon butyrate treatment (right side, hatched bars). Bars express mean values ± SD from three independent experiments, with single values shown as circles. Statistical testing was performed using a one‐way ANOVA with Tukey's multiple comparisons test, presenting only *p*‐values ≤0.07.

To determine the *C. albicans* burden in the intestinal compartment as well as its adhesion and invasiveness into the epithelial barrier, separate CFU assays from epithelial and endothelial washout and from the tissue layers were performed (Figure 5A). Non‐adhesive *C. albicans* within the epithelial washout represent planktonic fungal cells, which do not attach or are actively detached by shedding of self‐renewing epithelial cells. Invasive *C. albicans* hyphae within the epithelial tissue were analyzed after lysis of the tissue, and *C. albicans* deep tissue invasion was analyzed in the endothelial tissue and washout. Butyrate effectively prevented *C. albicans* invasion in the epithelial tissue under DSS inflammatory conditions (Figure 5B). We found no indication that the addition of DSS was rendering the growth of *C. albicans* (Figure S10). Butyrate further prevented deep tissue invasion of *C*. *albicans*, reaching the endothelial side. Interestingly, the growth of removed, non‐adhesive fungal colonies from the epithelial side was significantly increased by butyrate (Figure 5B). Considering the enhanced proliferation of epithelial cells, as quantified by Ki‐67 (Figure 3F), butyrate might play a critical role in self‐renewal of the epithelial tissue and the shedding of epithelial cells associated with the removal of adhesive *C. albicans* cells in DSS‐induced inflammation.

**FIGURE 6:**
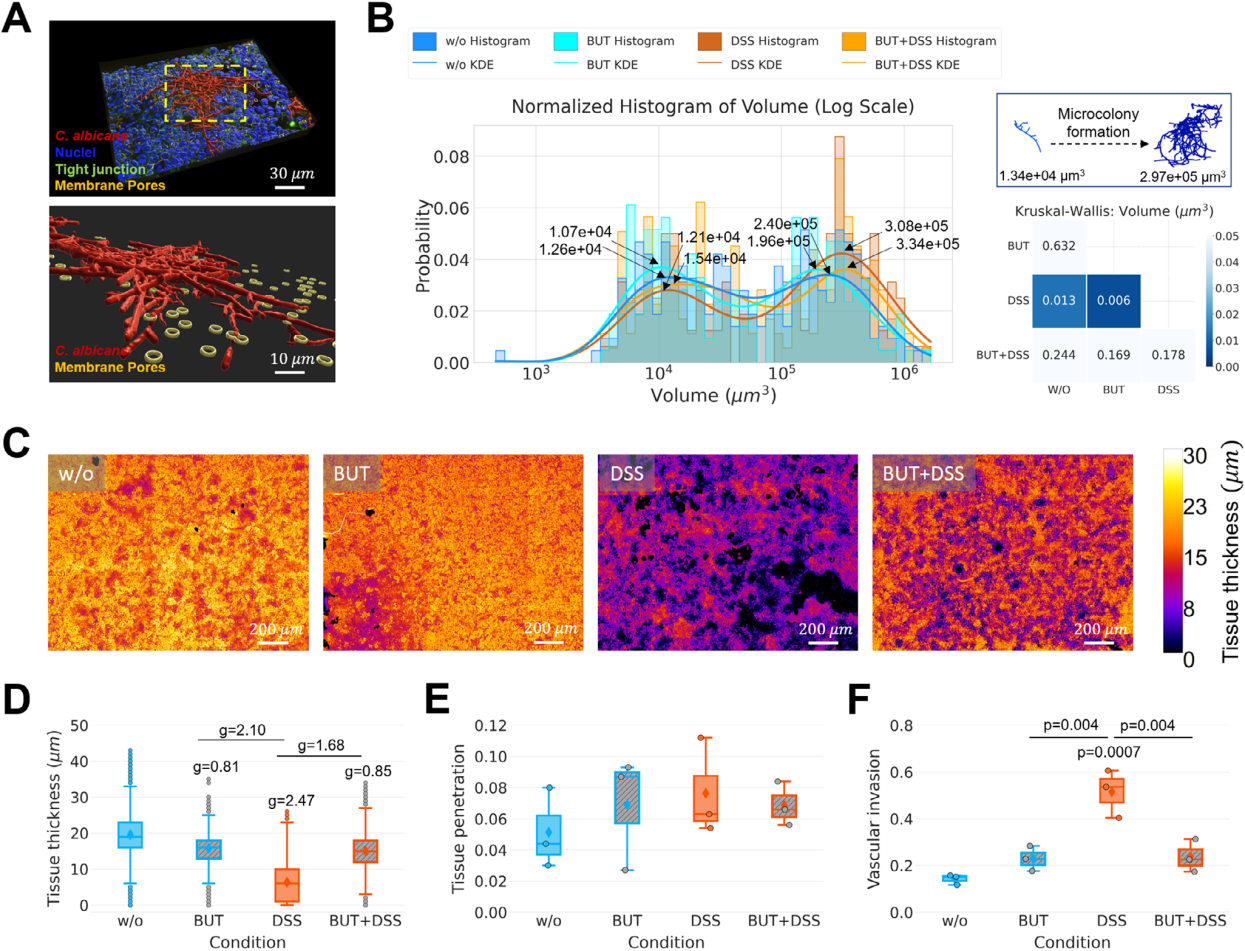
Image analysis of *C. albicans* infection in the CooC model. (A) 3D reconstructions of super‐resolution images of the intestinal epithelial cell layer 12 hpi with *C. albicans* SC5314 (upper image; *C. albicans*: red, nuclei: blue, tight junction: green, membrane pores: yellow, Scale bar, 30 µm). Magnified views of the intestinal and vascular layers reveal *C. albicans* microcolony exploiting membrane pores to translocate to the vascular side (lower image; *C. albicans*: red, membrane pores: yellow, Scale bar, 10 µm). (B) Normalized histogram of microcolony volumes on a log scale. The histogram displays the distribution of microcolony volumes, with the x‐axis representing volume (µm3) and the y‐axis representing the probability, where bar heights are normalized such that their sum equals 1. The Kernel Density Estimate (KDE) curve, generated using a Gaussian kernel, provides a smoothed, continuous representation of the data distribution. The representative hyphal structures corresponding to the *first histogram peak*, and the microcolonies associated with the *second peak*, are shown in the blue box in the top‐right corner. p‐values calculated using the Kruskal–Wallis test are presented in the matrix in the bottom‐right corner, with *p*≤0.05 considered statistically significant. (C) Epithelial tissue thickness heatmaps generated for an area of 4043.32 × 3049.06 µm^2^ per condition, with pixel‐level thickness values ranging from 0 to 30 µm. In the maps, brighter yellow hues correspond to increased epithelial thickness, and darker magenta tones denote thinner regions. (D) Epithelial tissue thickness distributions of 10 000 randomly selected points per image for each condition. (E) Tissue penetration by fungal microcolonies, quantified as the ratio of fungal microcolony volume within the epithelial tissue, normalized to account for variations in tissue thickness. No statistically significant differences were identified based on one‐way ANOVA (F). Vascular invasion, quantified as the ratio of the fungal microcolony volume within the vascular compartment to the total fungal volume. *p*‐values indicating statistical significance were derived from one‐way ANOVA with Tukey's post‐hoc test for multiple comparisons.

At 12 hpi, *C. albicans* microcolonies were expanding across the epithelial barrier and deeply invading the tissue to reach the endothelial compartment through membrane pores (Figure 6A). Volume distribution of C. albicans revealed a consistent bimodal pattern across all conditions, representing hyphal structures (smaller volume peak) and microcolonies (larger volume peak) (Figure 6B, blue box). While the non‐treated and butyrate‐treated groups exhibited similar distributions with peaks at ∼1.26 × 10^4^ µm^3^ and ∼2.40 × 10^5^ µm^3^ (non‐treated), and ∼1.07 × 10^4^ µm^3^ and ∼1.96 × 10^5^ µm^3^ (butyrate‐treated), DSS treatment shifted the distribution toward larger microcolonies. The primary peaks for DSS are observed at 1.21 × 10^4^ µm^3^ and 3.08 × 10^5^ µm^3^, with a higher probability for the larger colonies (0.0423) compared to the smaller colonies (0.0280). Butyrate treatment in the DSS group partially reversed this effect, with reduced probability for larger colonies (0.0360) and a volume distribution closer to the control and butyrate patterns, though the difference was not statistically significant (Figure 6B). The analysis of epithelial tissue thickness demonstrated a significant reduction in DSS‐treated samples compared to non‐treated and butyrate‐treated (Hedges’ g = 2.47 and 2.09, respectively). Butyrate treatment partially restored the epithelial thickness in the DSS‐treated, infected tissue (Hedges’ g = 1.68), remaining below control levels (Figure 6C,D).

The spatial analysis of host‐pathogen interaction revealed significantly increased invasion by fungal microcolonies reaching the endothelial side in DSS samples, correlating with the observed epithelial thinning. Butyrate treatment significantly reduced this invasiveness, likely by restoring epithelial tissue thickness and reinforcing its role as a physical barrier against fungal infiltration. Although the pattern of epithelial tissue invasion closely aligned with endothelial invasion, no statistically significant differences were observed in epithelial tissue invasion across experimental conditions (Figure 6E,F). These results highlight the ability of butyrate to mitigate DSS‐induced epithelial damage, reduce fungal invasion, and restore aspects of epithelial barrier integrity, supporting its therapeutic potential in colitis exacerbated by *C. albicans*.

### 3.4 Macrophages Modulate Butyrate‐Mediated Defence Against C. albicans

**FIGURE 7:**
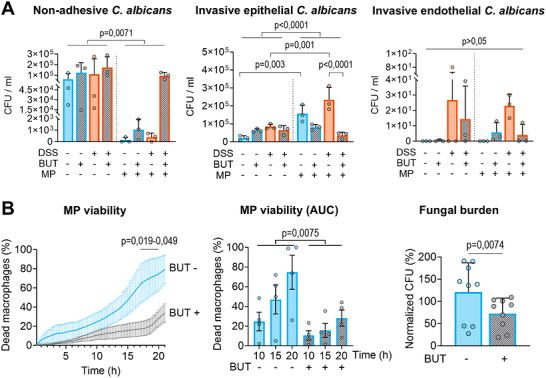
Macrophage‐dependent effects of butyrate on fungal burden, cytokine response, and macrophage viability in the CooC model. (A) Quantification of *C. albicans* colony‐forming units (CFU/mL) after 24 h of infection from (left) the epithelial waste (non‐adhesive fungi at luminal side), (middle) the epithelial tissue (invasive fungi in tissue), and (right) the endothelial compartment (translocated fungi to the vascular model side), in the presence or absence of macrophages (MΦ), DSS (1.5%), and butyrate (1 mm). Statistical testing was performed using a Two‐way ANOVA with Tukey's multiple comparisons test; exact *p*‐values are indicated. Data from three independent experiments are shown. (B) Left) Time‐resolved quantification of macrophage viability over 20 hpi, showing reduced cell death in butyrate‐treated conditions. Middle) Area under the curve (AUC) analysis of macrophage death for 10, 15, and 20 h timepoints. Right) Total fungal burden at endpoint, normalized to untreated controls. Statistical testing was performed for Middle) using a Two‐way ANOVA with Tukey's multiple comparisons test; for Right) using a two‐tailed *t*‐test; exact *p*‐values are indicated. Data from four independent experiments are shown. Bars represent mean ± SD; individual data points are shown as circles.

We further aimed to investigate the role of macrophages in the development of colitis in the intestine‐on‐chip model. Successful integration and long‐term maintenance of macrophages in the co‐culture was confirmed by CD68 immunofluorescence at experimental endpoints (Figure 1C,H), HLA‐DR/DQ/DP expression under flow (Figure S12), macrophage‐dependent cytokine release profiles (Table 4), identification of macrophage functional gene programs by bulk RNA‐sequencing (Figure S13D), and macrophage‐dependent modulation of fungal invasion (Figure 7A). First, we determined how macrophage presence alters baseline tissue characteristics in the intestine‐on‐chip model. We compared immunocompetent chips with macrophage‐free controls under untreated conditions. The presence of macrophages did not significantly affect LDH release, epithelial tissue thickness, endothelial AJCP expression (VE‐cadherin), or epithelial proliferation as measured by Ki‐67‐positive nuclei. However, it resulted in the formation of an improved epithelial AJCP network with higher E‐Cadherin volumes, which also contained more DAPI‐positive nuclei (Figure S11). These findings confirm that macrophages do not influence tissue homeostasis in the absence of inflammation‐modulating stimuli, such as DSS or butyrate, on the endothelial lining, but do help stabilize the formation and the cell growth of the epithelial tissue.

**TABLE 4:** Change of cytokine release relative to non‐treated gut‐on‐chip models without containing macrophages (in percent) (data from three independent experiments are shown).

	+ DSS	+ DSS/BUT	+ MΦ	+ DSS/MΦ	+ DSS/BUT/MΦ
IFN‐γ	66.9 ± 66.7	69,6 ± 60.0	381.4 ± 316.7	374.8 ± 149.1	205.7 ± 193.2
IL‐1β	79.5 ± 58.4	186.6 ± 131.0	100.6 ± 93.1	150.1 ± 130.1	84.5 ± 75.2
TNF‐α	77.6 ± 56.2	90.4 ± 13.1	503.7 ± 238.9	1300 ± 172.6	410.7 ± 203.8
MCP‐1	60.6 ± 52.5	24.8 ± 37.1	886.7 ± 741.5	271.8 ± 243.2	89.0 ± 74.3
IL‐6	71.4 ± 72.3	58.7 ± 20.0	106.4 ± 99.4	168.2 ± 33.4	47.3 ± 11.3
IL‐8	99.3 ± 91.1	105.2 ± 29.1	286.9 ± 243.4	645.7 ± 442.3	339.1 ± 188.6
IL‐10	86.6 ± 83.6	74.8 ± 71.5	91.9 ± 60.1	101.1 ± 79.4	62.6 ± 38.0
IL‐12p70	79.5 ± 69.1	89.6 ± 70.1	71.4 ± 31.1	90.0 ± 79.2	49.6 ± 13.31
IL‐17A	83.5 ± 73.1	74.0 ± 56.1	164.5 ± 112.1	196.6 ± 79.2	141.7 ± 107.0
IL‐18	67.8 ± 52.3	69.1 ± 35.8	82.4 ± 7.3	83.9 ± 33.1	78.2 ± 4.3
IL‐23	60.8 ± 33.1	71.1 ± 38.5	152.6 ± 118.1	185.8 ± 49.2	93.4 ± 61.66
IL‐33	66.6 ± 54.0	69.7 ± 64.1	111.2 ± 37.3	127.2 ± 60.2	65.1 ± 15.4

Next, we investigated the role of macrophages during *C. albicans* infection in the CooC model and the effects of butyrate in this process. We observed that butyrate treatment significantly impacted fungal colonization and epithelial invasion in a macrophage‐dependent manner. In the intestinal compartment, the number of non‐adhesive *C. albicans* cells showed a trend toward reduction when macrophages were absent. However, when treated with DSS and butyrate, the presence of macrophages did not further reduce the non‐adhesive fungi in the medium compared to macrophage‐free tissues (Figure 7A, left). The number of fungi invading the epithelial tissue was increased in the presence of macrophages under both untreated and DSS‐treated conditions (Figure 7A, middle). However, butyrate treatment substantially reduced fungal invasion in the epithelial layer in macrophage‐containing models, demonstrating that butyrate restricts epithelial barrier breach by modulating macrophage function and epithelial integrity. Notably, butyrate treatment also substantially reduced fungal translocation in macrophage‐containing models (Figure 7A, right).

Cytokine analysis revealed a general trend toward increased inflammatory cytokine release in the presence of macrophages across all conditions. In particular, TNF, MCP‐1, and IL‐8 were markedly elevated, suggesting an activated macrophage response during infection (Table 4). Butyrate co‐treatment modulated this cytokine profile by preventing an excessive, potentially tissue‐damaging immune response, while preserving a level of pro‐inflammatory signaling which is sufficient for an effective defense against fungal invasion in the presence of macrophages. Live‐cell imaging further showed that butyrate improved macrophage viability during infection with *C. albicans* (Figure 7B, left, Videos SV1). This effect was statistically supported by reduced area under the curve (AUC) values for macrophage death in the butyrate‐treated condition (Figure 7B, middle). The increased macrophage viability upon butyrate treatment was found to be associated with an inhibition of fungal outgrowth (Figure 7B, right), suggesting that increased macrophage resilience induced by butyrate enhances fungal clearance.

**FIGURE 8:**
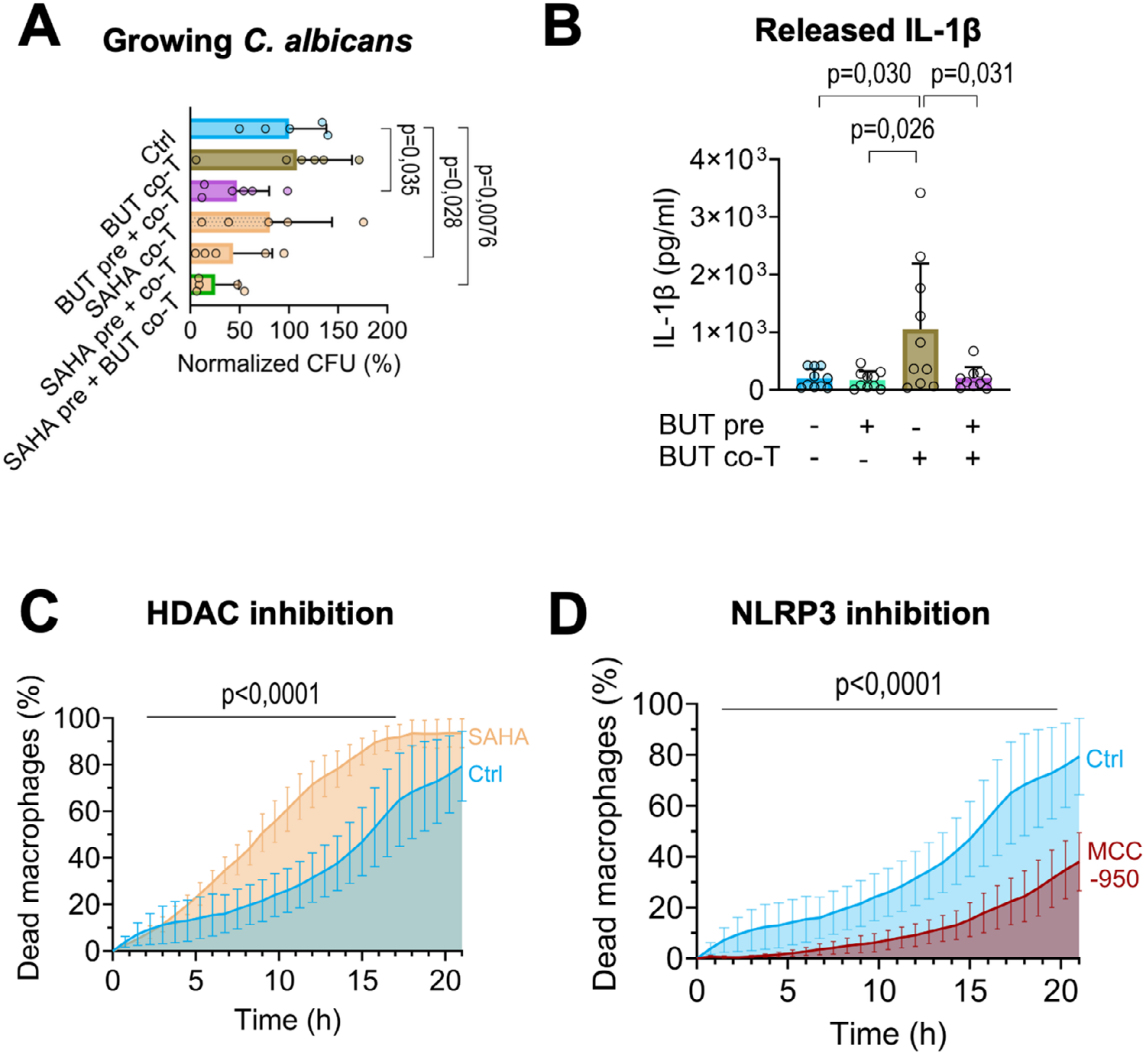
HDAC inhibition by SAHA and butyrate modulates fungal burden, cytokine release, and inflammasome‐dependent macrophage viability during *C. albicans* infection. (A) Colony‐forming unit (CFU) analysis shows reduced fungal burden in macrophage cultures following 24 h pretreatment (pre) with SAHA or butyrate (BUT), compared to simultaneous co‐treatment (co‐T) conditions. (B) IL‐1β secretion quantified by multiplex cytokine with butyrate pretreatment (BUT pre) vs. co‐treatment (BUT co‐T) (C,D). Time‐resolved viability analysis of macrophages infected with *C. albicans* under HDAC inhibition (SAHA), and NLRP3 inflammasome inhibition (MCC‐950). Data represent mean ± SD; statistical significance determined by two‐way ANOVA or unpaired two‐tailed t‐tests as indicated).

We next analyzed whether the protective activity of butyrate can be attributed, at least in part, to its known epigenetic mechanism of action via histone deacetylase (HDAC) inhibition. Butyrate is a well‐established inhibitor of HDAC activity, thereby modulating gene expression in immune cells [35]. To investigate the role of HDAC‐dependent immunomodulatory effects of butyrate in the context of fungal infection, we tested the synthetic HDAC inhibitor SAHA (suberoylanilide hydroxamic acid) in our model and assessed its effects on fungal clearance and cytokine responses to infection. SAHA pretreatment (pre) for 24 h before infection mimicked the protective effect of butyrate, significantly reducing *C. albicans* burden in macrophage cultures (Figure 8A). This effect depended on timing, as co‐treatment immediately at the time of infection without preincubation (co‐T) failed to produce the same outcome, suggesting that transcriptional reprogramming prior to pathogen contact is required, which is consistent with the known HDAC‐inhibiting mechanism of SAHA. A similar pattern was observed with butyrate, though the antifungal effects were less pronounced.

In terms of cytokine regulation, SAHA pretreatment exerted a broad anti‐inflammatory effect across multiple cytokines (Table 4), including IL‐1β, TNF, and IL‐6. Butyrate also reduced pro‐inflammatory cytokine release, but this effect was generally weaker and was observed statistically significantly only for IL‐1β (Table 5). Notably, butyrate pretreatment 24 h prior to infection significantly suppressed IL‐1β secretion, while its simultaneous co‐administration with *C. albicans* at the time of infection enhanced IL‐1β release (Figure 8B). These context‐dependent outcomes align with recent findings that SCFAs, particularly butyrate, can activate the NLRP3 inflammasome in macrophages primed with Toll‐like receptor ligands. Mechanistically, this effect is HDAC‐dependent, bypassing pyroptosis and inducing a hyperactivated cytokine secretion phenotype [36]. In contrast to butyrate, SAHA co‐treatment was associated with an elevated macrophage cell death upon encounter with *C. albicans* (Figure 8C). This highlights the importance of timing in pathogen detection and the subsequently induced antifungal immune response. Our findings indicate that pretreatment with butyrate triggers a tolerogenic transcriptional program that attenuates inflammasome activation upon exposure to *C. albicans*. In contrast, simultaneous exposure to butyrate and *C. albicans* may synergize to activate the NLRP3 inflammasome.

**TABLE 5:** **TABLE 5** Cytokine release profiles from macrophages treated with SAHA (fold change in percent compared to non‐infected control). Data represent mean ± SD; statistical significance determined by two‐way ANOVA with *p*<0,0001 (data from four independent experiments are shown).

	+ SAHA pre	+ SAHA co‐T	+ SAHA pre + co‐T
IL‐1β	10972 ± 8637	16729 ± 25999	2330 ± 436.8
IFN‐α2	140.6 ± 34.8	93.0 ± 16.3	54.7 ± 38.9
IFN‐γ	177.4 ± 62.4	26.3 ± 8.0	13.5 ± 18.0
TNF‐α	2089 ± 224.0	727.0 ± 625.7	25.9 ± 18.5
IL‐6	165.2 ± 33.9	77.1 ± 76.7	14.4 ± 27.2
IL‐8	113.5 ± 15.8	113.5 ± 9.6	104.3 ± 11.9
IL‐10	394.5 ± 298.1	16.6 ± 13.7	4.5 ± 0,5
IL‐12p70	450.4 ± 412.7	81.1 ± 48.1	27.5 ± 35.7
IL‐17A	797.7 ± 421.3	177.2 ± 171.4	76.2 ± 12.8
IL‐18	676.6 ± 388.6	393.9 ± 305.2	185.2 ± 313.2
IL‐23	2550 ± 2770	82.2 ± 60.9	30.3 ± 46.9
IL‐33	237.5 ± 81.0	76.1 ± 61.1	38.1 ± 55.0

**TABLE 6:** **TABLE 6** Cytokine release profiles from macrophages treated with butyrate (fold change in percent compared to non‐infected control). Data represent mean ± SD; statistical significance determined by two‐way ANOVA (data from three independent experiments are shown).

	+ BUT pre	+ BUT co‐T	+ BUT pre + co‐T
IFN‐α2	121.7 ± 40.0	93.0 ± 16.3	86.8 ± 30.7
IFN‐γ	127.9 ± 52.7	26.3 8±.0	104.2 ± 33.1
TNF‐α	9370 ± 6647	727.0 ± 963.5	3009 ± 5197
IL‐6	123.3 ± 32.9	77.1 ± 7±6.7	39.1 ± 47.9
IL‐8	101.3 ± 9.8	113.5 ± 7.4	94.6 ± 7.5
IL‐10	828.5 ± 964.7	16.6 ± 13.7	64.5 ± 6.8
IL‐12p70	112.2 ± 45.6	81.1 ± 48.1	61.1 ± 15.1
IL‐17A	393.4 ± 386.1	177.2 ± 171.4	115.4 ± 83.7
IL‐18	1920 ± 1534	393.9 ± 305.2	820.0 ± 124.7
IL‐23	439.2 ± 287.7	82.2 ± 60.6	173.1 ± 152.9
IL‐33	254.9 ± 159.7	76.1 ± 61.1	157.0 ± 142.3

To test this further, we pharmacologically inhibited inflammasome activity in macrophages upon contact with *C. albicans* using MCC‐950 (a specific NLRP3 inhibitor [37]). MCC‐950 improved macrophage survival during fungal infection (Figure 8D), confirming a key role for NLRP3 inflammasome activation in *C. albicans*‐induced macrophage cell death and its modulation by SCFA signaling.

## 4 Discussion

### 4.1 DSS‐Based IBD‐on‐Chip Model Replicates Hallmarks of IBD

A central feature of the intestine‐on‐chip model is continuous medium perfusion, which we show to be a prerequisite for establishing a physiologically relevant, immunocompetent tissue environment. Bulk RNA sequencing comparing briefly and continuously perfused cultures revealed that continuous flow induced widespread transcriptomic reprogramming, affecting over 4,000 genes. Gene set enrichment analysis identified immune activation and antigen presentation as the most prominently enriched functional programs under continuous flow. The upregulation of MHC class II assembly pathways under flow is consistent with the maturation of monocytes into professional antigen‐presenting cells, as tissue‐infiltrating monocytes progressively acquire MHC class II expression during differentiation into macrophages [38, 39]. The concurrent downregulation of MHC class I antigen processing pathways may reflect reduced cellular stress responses under physiological flow conditions. This was confirmed by the coordinated upregulation of macrophage genes spanning all functional categories, from identity markers and pro‐inflammatory mediators to anti‐inflammatory regulators and phagocytic effectors. This balanced transcriptional profile, encompassing both pro‐inflammatory and anti‐inflammatory gene programs, suggests that continuous perfusion promotes a more differentiated macrophage state compared to static culture, with features that partially reflect the dual capacity of tissue macrophages to maintain homeostasis while retaining immune competence. The enrichment of fatty acid metabolic pathways under continuous flow further suggests enhanced metabolic competence of the perfused tissue, which is particularly relevant for the processing of microbiota‐derived short‐chain fatty acids such as butyrate. These transcriptomic findings complement our immunofluorescence data showing improved endothelial (CD31) and epithelial (ZO‐1) junction networks and maintained HLA‐DR/DQ/DP‐positive macrophages under continuous perfusion, and are consistent with published gut‐on‐chip transcriptomic studies [40]. These data establish that continuous perfusion is essential for generating the immunocompetent baseline on which the subsequent colitis and infection experiments are built (Table 6).

Based on this, we established a human CooC model that reproduces key features of IBD. Short‐term exposure to 1.5% DSS for 48 h resulted in a sustained loss of epithelial barrier function, a robust pro‐inflammatory cytokine response, and the increased susceptibility of the intestinal tissue to invasion by *C. albicans* upon infection. These effects mirror important pathological hallmarks of IBD. These outcomes align with in vivo DSS‐induced colitis models and prior gut‐on‐chip systems, confirming the relevance of our model system [41]. Incorporating human macrophages into the model added an immune component to the epithelial‐microbial crosstalk, capturing the intercellular dynamics that drive intestinal inflammation. DSS exposure for 24 h reduced CD68 signal intensity in monocyte‐derived macrophages without corresponding LDH release or loss of macrophage nuclei, indicating phenotypic modulation rather than macrophage depletion. This interpretation is in line with previous findings from DSS colitis models, where inflammatory activation remodels CD68 macrophage marker expression without reducing macrophage abundance [42]. Likewise, LPS‐tolerant macrophages exhibit reduced inflammatory marker expression through transcriptional reprogramming despite preserved survival [43]. Consistent with reports from murine DSS colitis, the presence of *C. albicans* in our model exacerbated tissue injury and inflammation [2, 44]. This underscores the clinical observation that fungal overgrowth can worsen IBD severity [6, 12, 45]. The increased susceptibility of DSS‐treated tissue to *C. albicans* invasion may arise from compromised epithelial architecture and reduced tissue resilience, which lowers the physical threshold for hyphal invasion and nutrient acquisition. Macrophages were found to enhance *C. albicans* invasion into the epithelial tissue during fungal infection. This effect is likely mediated by macrophage‐secreted pro‐inflammatory cytokines that drive tight junction disruption and increased barrier permeability. Moreover, the inflammatory environment may influence macrophage function and impair their antifungal capacity. These factors create a permissive niche for fungal expansion in the inflamed mucosa. Alternatively, *C. albicans* may sense inflammatory signals from macrophages as cues to enhance its pathogenicity [46], as shown previously in response to alpha‐1 antitrypsin [47].

### 4.2 Butyrate Enhances Epithelial Regeneration and Barrier Function

We recapitulated the protective effect of butyrate on the intestinal epithelium during colitis and fungal tissue invasion, aligning with previous results from animal studies and clinical observations. Butyrate preferentially stabilized the adherens junction complex, consistent with its established role as an HDAC inhibitor that promotes membrane localization of E‐cadherin [48, 49]. In contrast, the tight junction protein ZO‐1 is regulated through additional pathways, including inflammatory cascades, that might be deregulated during *C. albicans* hyphal invasion and DSS‐mediated injury. Several studies show that butyrate can support ZO‐1 maintenance under mild stress, yet fails to prevent ZO‐1 loss during severe inflammatory or infectious challenge [50, 51]. Our findings thus align with this differential vulnerability, with butyrate preserving E‐cadherin expression under infection conditions, whereas ZO‐1 network integrity remains diminished. Nonetheless, the partial stabilization of adherens junctions and enhanced epithelial renewal likely contribute to the observed trend toward improved epithelial barrier function despite incomplete ZO‐1 rescue.

In murine models, sodium butyrate supplementation has been shown to alleviate symptoms of colitis. For instance, in a TNBS‐induced colitis model, sodium butyrate maintained gut epithelial barrier integrity and reduced intestinal inflammation [52]. Furthermore, in DSS‐induced colitis models, pretreatment with butyrate was found to restore tight‐junction protein formation and to reduce epithelial permeability [53]. Another study demonstrated that sodium butyrate alleviated DSS‐induced colitis in mice by modulating gut microbiota and maintaining intestinal barrier integrity [54]. Clinically, butyrate has shown potential to manage IBD by regulating immune function, epithelial barrier function, and intestinal homeostasis, suggesting that butyrate supplementation could be beneficial for reducing inflammation and maintaining remission in IBD patient [49, 55, 56]. Our model system provides additional mechanistic insights highlighting the detrimental effects of *C. albicans* tissue invasion during colitis, and cell‐type‐specific pleiotropic functions of butyrate, which acts distinctly on epithelial cells and macrophages, providing two synergistic layers of defense against *C. albicans*. This dual action underscores the versatility of butyrate as a potential therapeutic agent in IBD contexts where disease severity is exacerbated by *C. albicans*, particularly where such complexity is challenging to address in animal models.

Butyrate supplementation stimulated intestinal epithelial cell proliferation and accelerated epithelial turnover. This may have promoted the shedding of infected cells from the surface. Similar to shedding induced by the probiotic *L*. rhamnosus [57], this rapid self‐renewal may have physically dislodged invading *C. albicans* and thereby reduced the fungal burden on the mucosa. The enhanced epithelial regeneration was accompanied by improved barrier integrity. In the CooC model, butyrate treatment increased apical junctional protein E‐cadherin under DSS‐induced stress, curtailing paracellular permeability and preventing fungal invasion into deeper tissues. These findings are consistent with previous studies demonstrating that butyrate can restore epithelial junctional complexes and fortify barrier function during colitis [2, 6, 58, 59]. Improved barrier maintenance is crucial in IBD, as even low‐grade dysbiosis can lead to pathogen invasion when the barrier integrity is compromised. By stabilizing the epithelial barrier, butyrate creates an environment less permissive to opportunistic invasion. Previous studies support this concept, in which exogenous butyrate or butyrate‐producing bacteria help ameliorate colitis by reinforcing mucosal defenses [58, 59]. Our CooC data extend these observations, illustrating that bolstering the epithelium's intrinsic defenses with butyrate can be an effective strategy to limit fungal invasion in an IBD setting.

### 4.3 Immunomodulatory Effects of Butyrate on Macrophages

Beyond the epithelium, butyrate provided a second layer of protection against fungal intestinal barrier invasion by modulating macrophage function in the inflamed intestinal tissue model. We observed that macrophages exposed to butyrate became more resilient to *C. albicans*‐induced lysis, displaying a more efficient fungal clearance. Importantly, this improved antimicrobial activity did not come at the expense of excessive inflammation. In contrast, butyrate‐treated macrophages in our model released lower levels of pro‐inflammatory cytokines (such as TNF and IL‐6) and higher levels of the regulatory cytokine IL‐10, indicative of a shift toward an anti‐inflammatory or tolerant M2‐like phenotype. In line with this observation, recent studies report that butyrate can recalibrate macrophage polarization and dampen hyperinflammatory responses while preserving pathogen‐killing functions. In the presence of butyrate, macrophages were shown to upregulate antimicrobial pathways (including autophagy‐related defenses and microbicidal peptides) without a concomitant increase in inflammatory cytokines, an effect mediated through histone deacetylase 3 (HDAC3) inhibition [3]. This balance is critical in the context of IBD, in which hyperinflammatory responses can damage host tissue, whereas an insufficient response permits uncontrolled infection [60]. Butyrate achieved an optimal equilibrium in our model, restraining excessive inflammation yet maintaining effective antifungal immunity. This dual immunomodulatory role aligns with the emerging understanding that butyrate helps restore immune homeostasis in the gut during colitis [3, 61]. The presence of macrophages also limited epithelial damage in butyrate‐treated models despite ongoing infection, thus highlighting how an immune modulatory effect of butyrate can protect tissue integrity.

### 4.4 Butyrate Limits Fungal Morphogenesis and Burden

We further capitalized on extensive morphometric analyses of *C. albicans* within the chip to understand how butyrate impacts fungal behavior in colitis. In the context of DSS‐treated chips, *C. albicans* formed filaments and invated the already compromised epithelium, consistent with its pathogenic transition under inflammatory conditions. Strikingly, butyrate significantly blunted *C. albicans* tissue invasion. The fungus showed reduced adhesion to the epithelium, stunted hyphal elongation, and minimal tissue invasion in butyrate‐treated conditions. This resulted in a lower fungal load and less epithelial damage. These observations are in agreement with known antifungal effects of SCFAs. Butyrate, at physiological concentrations, has been shown to directly inhibit *C. albicans* yeast growth and hyphal development, and even suppress biofilm formation in vitro [5]. Mechanistically, one plausible explanation is that butyrate forces *C. albicans* to alter its metabolism. *C. albicans* can utilize fatty acids like butyrate as carbon sources, but doing so requires a metabolic shift to less fermentative pathways, such as the glyoxylate cycle [15]. This metabolic rerouting may attenuate the fungus's virulence, as it diverts resources away from rapid growth and tissue damage toward survival mode. Indeed, it is known that growth on alternative carbon sources can constrain *C. albicans* tissue invasion and virulence factor expression [62]. Remarkably, this metabolic shift occurred despite the ample glucose availability in our chip model, where *C. albicans* would typically favor rapid, glucose‐driven growth. Additionally, butyrate and related SCFAs might affect the fungal cell surface and quorum sensing, limiting its capacity to form hyphae and invade host cells [15]. The net effect in our model might be a synergistic protection: while butyrate‐fortified epithelium shed fungi and kept them at bay, the altered fungal morphology and metabolism in the presence of butyrate made *C. albicans* less harmful and easier to clear. This two‐pronged reduction in fungal fitness is particularly relevant to colitis, as uncontrolled fungal proliferation has been linked to worsened colonic inflammation and barrier disruption [5]. By suppressing *C. albicans* colonization, butyrate can support to break the vicious cycle of dysbiosis and mucosal injury in IBD.

### 4.5 HDAC Inhibition and Inflammasome Modulation by Butyrate

A noteworthy aspect of butyrate action is its role as an HDAC inhibitor, which emerged as a key mechanism in shaping the immune response. HDAC inhibition can broadly reprogram gene expression in macrophages, as seen for butyrate in the promotion of an antimicrobial, inflammation‐dampened phenotype via HDAC3 blockade [3]. One downstream consequence of this reprogramming is the modulation of the NLRP3 inflammasome pathway and pyroptotic cell death. Butyrate was further shown to reduce expression of *Il6*, *Nos2*, and *Il12* genes without affecting *Tnfa* or Ccl2 [63]. This epigenetic modulation likely underlies its capacity to attenuate inflammasome activation.

In our CooC containing macrophages treated with butyrate, we found no signs of excessive inflammasome activation (such as overt IL‐1β‐driven pyroptosis) despite the presence of *C. albicans*, suggesting that butyrate kept inflammasome activity in check. Interestingly, in our CooC model, IL‐1β levels after stimulation were generally lower than in macrophage monoculture. This observation suggests that epithelial‐macrophage crosstalk during differentiation within the chip environment may modulate macrophage plasticity, steering them toward a phenotype less prone to inflammasome activation and IL‐1β secretion. Such intercellular interactions have been shown to influence macrophage behavior. Epithelial‐derived signals can prime macrophages to adopt anti‐inflammatory or tissue‐repair phenotypes, thereby attenuating inflammasome activation and subsequent IL‐1β release [64]. This aligns with findings where epithelial‐macrophage interactions modulate immune responses [65], potentially contributing to the controlled inflammatory milieu observed in our chip model.

Recent in vivo work further supports an anti‐pyroptotic effect of butyrate. Fan et al. demonstrated that oral sodium butyrate markedly alleviated DSS‐colitis in rats by inhibiting macrophage pyroptosis, through reduction of mitochondrial ROS formation and suppression of NF‐κB signaling [58]. Butyrate increased tight junction protein expression in parallel, an outcome associated with the prevention of inflammasome‐induced epithelial damage. Thus, HDAC‐mediated inflammasome restraint by butyrate may be critical in protecting the gut mucosa from collateral inflammatory injury. On the other hand, the influence of butyrate on inflammasome pathways can be context‐dependent. Interestingly, one study found that while butyrate alone did not trigger IL‐1β release, its presence potentiated inflammasome activation when macrophages were concurrently stimulated with specific bacterial ligands (e.g., lipoteichoic acid from *Enterococcus faecalis*) [66]. In that scenario, butyrate‐mediated HDAC inhibition lowered the threshold for caspase‐1 activation and boosted IL‐1β secretion, yet notably without causing pyroptotic cell lysis. This suggests that butyrate can fine‐tune inflammasome responses by dampening excessive pyroptosis or enhancing a controlled IL‐1β response, depending on the immune context and signals present. In our *C. albicans* colitis infection model, the prevailing effect of butyrate was protective. Butyrate tempered undue inflammation while still permitting effective antifungal defense, as evidenced by successful pathogen clearance with minimal host tissue damage.

Butyrate pretreatment induces a tolerogenic macrophage phenotype and markedly improved macrophage viability upon *C. albicans* exposure, whereas co‐treatment with SAHA at the time of infection reduced viability. Stimulation time‐dependent effects of butyrate (pretreatment vs co‐stimulation) may be understood as a context‐driven immune response. Under homeostatic conditions, macrophages residing in the gut are continuously exposed to microbiota‐derived butyrate, which dampens their activation and inflammasome responsiveness, an adaptive mechanism to prevent excessive inflammation in a microbe‐rich environment. Occasional encounters with fungal pathogens such as *C. albicans* in this context may thus be effectively contained by viable, tolerized macrophages. In contrast, during dysbiosis, the loss of butyrate and concurrent fungal overgrowth shift the immune strategy. Here, macrophages encountering *C. albicans* in the absence of tolerogenic signals may interpret the situation as a breach of barrier integrity, triggering inflammasome activation and pyroptosis to recruit neutrophils and contain the threat.

While simultaneous exposure of unconditioned macrophages to butyrate and *C. albicans* may be rare under physiological conditions, a similar situation might arise during therapeutic interventions, e.g., administration of butyrate or butyrate‐producing probiotics in patients with ongoing fungal infections under dysbiotic conditions, such as in IBD. In such scenarios, the convergence of microbial PAMPs and butyrate may lead to excessive inflammasome activation, underscoring the importance of timing and context in modulating antifungal immunity. This nuanced immunomodulation highlights the therapeutic potential of targeting HDAC pathways in IBD. Our findings align with earlier reports showing that butyrate renders colonic lamina propria macrophages less responsive by downregulating pro‐inflammatory gene expression [63].

In contrast to butyrate, we found that SAHA co‐treatment accelerated macrophage cell death upon fungal contact. This differential effect may be attributed to the distinct mechanisms by which these compounds modulate immune responses. The distinct selectivity and pharmacokinetics of these HDAC inhibitors can explain this divergence. Butyrate primarily inhibits class I HDACs (HDAC1–3) and class IIa HDACs with minimal activity against HDAC6 [67], thereby promoting a pro‐survival, antimicrobial transcriptional program in macrophages [3]. In contrast, SAHA is a broad‐spectrum inhibitor targeting HDAC1–3, 6, 8, 10, and 11, which leads to widespread histone and tubulin hyperacetylation (via HDAC6 inhibition) and broad transcriptional alterations [68]. While butyrate rapidly engages GPR signaling and inhibits HDACs to establish a tolerogenic state, SAHA acts predominantly via epigenetic remodeling and requires more time to exert transcriptional changes. Pre‐treatment with SAHA, rather than simultaneous exposure, might confer protective effects by reprogramming macrophage transcriptional responses prior to fungal challenge. This highlights the importance of timing in HDAC inhibitor‐mediated immune modulation and suggests that SAHA may be beneficial in prophylactic rather than acute therapeutic settings. Future studies should explore whether SAHA pre‐conditioning promotes macrophage survival and antifungal competence, and how this differs from the rapid, context‐dependent effects observed with butyrate. Leveraging butyrate or HDAC inhibitors may bolster mucosal defenses (both epithelial and myeloid) against opportunistic infections like candidiasis, without provoking the detrimental inflammation that characterizes active IBD.

Several aspects of the present findings extend specific observations from prior animal models, clinical studies, and in vitro systems. First, murine DSS colitis studies established that *C. albicans* exacerbates intestinal inflammation in a strain‐specific manner, with severity linked to the capacity to cause tissue damage [12]. Ost et al. demonstrated in gnotobiotic mice that adaptive immunity, particularly anti‐hyphae antibodies, is required to maintain *C. albicans* gut colonization [69], while Leonardi et al. identified CX3CR1^+^ mononuclear phagocytes as key mediators of intestinal antifungal immunity [70]. However, these murine models rely on hosts that are not natural carriers of *C. albicans*, and the individual contributions of macrophage‐fungus interactions vs. epithelial barrier effects cannot be readily dissected in vivo. Using the immunocompetent CooC model, we demonstrate that macrophage‐*C. albicans*‐interaction is a minimum requirement for fungal exacerbation of colitis‐like tissue damage (Figure 7A), and this process is strongly modulated by the microbiota‐derived metabolite butyrate. Butyrate exerts colonization resistance against *C. albicans* through multiple mechanisms described in separate experimental systems. Savage et al. showed that Clostridia‐derived butyrate maintains epithelial hypoxia via PPAR‐γ signalling, restricting *C. albicans* oxygen availability and growth in the murine gut [7]. Guinan et al. demonstrated that SCFAs at in vivo murine cecal concentrations directly inhibit *C. albicans* growth and filamentation in vitro [5]. Meanwhile, the effects of butyrate on host cells have been examined by others on the epithelial side, with HDAC inhibition and E‐cadherin stabilization characterized in murine DSS colitis [52, 53] and in cell culture systems [48, 50]. Butyrate was further shown to reprogram macrophage function toward antimicrobial and anti‐inflammatory phenotypes through HDAC inhibition [3, 63]. Nguyen et al. further demonstrated that sodium butyrate enhances macrophage antifungal activity by augmenting reactive oxygen species and nitric oxide production [71]. In our multicellular co‐culture system, these epithelial and macrophage effects occur simultaneously and are interconnected, as butyrate stabilizes epithelial adherens junctions and promotes epithelial renewal, but concurrently reprograms macrophage responses to limit fungal invasion while preserving antifungal capacity (Figure 7). Notably, at the 1 mm concentration used in our model, butyrate did not affect *C. albicans* growth or morphology in isolation, consistent with the lower end of physiological colonic concentrations and in contrast to the higher concentrations used by Guinan et al. [5]. This distinguishes the host‐mediated dual protective mechanism observed here from direct antifungal effects. Recent work has revealed that the immunomodulatory effects of butyrate are context‐dependent and can be paradoxical. Wang et al. showed that in the presence of TLR agonists, butyrate activates the NLRP3 inflammasome in human macrophages through HDAC‐dependent loss of cFLIP and IL‐10, triggering caspase‐8‐dependent IL‐1β release [36]. Park et al. reported similar inflammasome potentiation by butyrate in the context of bacterial products [66]. Our data are consistent with this paradoxical enhancement when butyrate is applied simultaneously with infection. However, butyrate pre‐exposure establishes a tolerogenic macrophage program prior to fungal challenge, suppressing NLRP3 inflammasome activation and protecting macrophage viability. The HDAC inhibitor SAHA phenocopied this pre‐treatment effect, supporting a time‐sensitive HDAC/NLRP3 axis rather than nonspecific immunosuppression. This timing dependency in butyrate‐mediated immune modulation has not been systematically addressed in prior studies of fungal infection.

Furthermore, quantitative 3D microcolony invasion metrics, including projected area, hyphal compactness, depth‐of‐penetration distribution, and surface density, provide spatially resolved endpoints that are not captured by CFU enumeration alone. In murine models, identifying the precise sites of *C. albicans* mucosal invasion remains inherently challenging [17]. The image‐analysis pipeline applied here resolves how butyrate simultaneously increases luminal clearance and prevents deep tissue penetration at the single‐microcolony level. With this, our data primarily support the preventive effect of butyrate on intestinal conditioning. Future investigations may assess post‐injury dosing, SCFA analogues, and HDAC‐modulating adjuncts as potential therapeutic strategies.

Compared to the foundational characterization of the intestine‐on‐chip model in Maurer et al. [16], which investigated probiotic–pathogen interactions and basic macrophage phenotyping via surface markers, the present study addresses a fundamentally different biological question on how the metabolic end‐products of a healthy microbiota promote immune tolerance and mucosal resilience during fungal exacerbation of colitis. The cell‐type‐specific and temporally dependent roles of butyrate in this dynamic host‐pathogen‐metabolite interplay provide mechanistic insights that complement findings from animal models and simpler in vitro systems. However, we recognize the reductionist nature of the CooC model, which captures only selected IBD‐relevant injury and cytokine features, with modest shifts in TNF‐α and IL‐1β observed under acute DSS/LPS stimulation. While DSS provides a microbiota‐independent epithelial injury that we leverage to isolate host responses, in vivo dysbiosis can amplify inflammation and diminish colonization resistance. Future iterations will integrate a defined synthetic microbiota to capture bacterial‐fungal competition and metabolite gradients under flow and to test whether the microcolony/penetration signatures observed here generalize across dysbiotic states. Therapeutically, restoring luminal butyrate, either via diet, probiotics, or direct supplementation, serves as a two‐pronged strategy to reinforce the epithelial‐immune interface in ulcerative colitis and to reduce the risk of opportunistic fungal exacerbations.

## Author Contributions

M.A. and M.W. performed the organ‐on‐chip experiments and microscopy. P.A. and Z.C. carried out image processing and morphometric analysis. R.A.‐R. and A.D. contributed to fungal burden assays and cytokine profiling. V.W., A.F., and M.I.A.H. supported cell culture and immunostaining. S.M. and O.H. provided resources and methodological input. B.H. provided resources, including staff funding, the *Candida albicans* strain, and expert interpretation of fungal infection data. M.T.F. advised on data analysis workflows and developed image quantification tools. A.S.M. wrote the manuscript with input from all co‐authors. A.S.M. and M.G. conceived the project. A.S.M. supervised the overall study. Y.B. performed analysis of bulk RNA‐seq data. All authors discussed the results and approved the final version of the manuscript.

## Funding

M.S.G. was supported by the German Research Foundation (Deutsche Forschungsgemeinschaft—DFG) Emmy Noether Program (project no.434385622/GR 5617/1‐1). A.D. was supported by an Exploration Grant of the Boehringer Ingelheim Foundation (BIS) to M.S.G. This research was further supported by the Free State of Thuringia and co‐funded by the European Union—Project‐ID 2023 FGI 0004. “A Live broadcast of the interactions between host and fungal pathogens” to M.S.G. and A.D., R.A.R., A.S.M., M.T.F. and B.H. acknowledge funding by DFG Cluster of Excellence “Balance of the Microverse” under Germany's Excellence Strategy—EXC 2051, Grant Number: 390713860. A.S.M. was further supported by the BMFTR program Photonics Research Germany (FKZ: 13N15713, 13N15716), which is integrated into the Leibniz Center for Photonics in Infection Research (LPI). The LPI initiated by Leibniz‐IPHT, Leibniz‐HKI, UKJ, and FSU Jena is part of the BMBF national roadmap for research infrastructures. This work was further funded by DFG within the Collaborative Research Center “PolyTarget” to M.T.F. (SFB 1278 – project ID 316213987, subproject Z01). M.A. and M.W. were supported by a Scholarship from the Interdisciplinary Center of Clinical Research of the Jena University Hospital (Interdisziplinäres Zentrum für Klinische Forschung, IZKF Jena).

## Conflicts of Interest

The authors declare no conflicts of interest.

## Supporting information




**Supporting File**: smll73749‐sup‐0001‐SuppMat.docx.

## Data Availability

The data discussed in this manuscript have been deposited in NCBI's Gene Expression Omnibus and are accessible through GEO Series accession number GSE325894.
