# Supplementary material for: Human Colitis‐on‐Chip Model Reveals Dual Roles of Butyrate in Epithelial and Macrophage Defense Against Candida albicans Tissue Invasion
Source: Small. 2026 May 15;22(37):e00074. doi: 10.1002/smll.202600074 (PMC13325699; doi:10.1002/smll.202600074)
Supplement: Supplementary file 1 — Supporting File: smll73749‐sup‐0001‐SuppMat.docx. [file SMLL-22-e00074-s001.docx]

**Supplementary Material for:**

**Human Colitis-on-Chip Model Reveals Dual Roles of Butyrate in Epithelial and Macrophage Defense Against *Candida albicans* Tissue Invasion**

**Authors**

Manuel Allwang^1,2^, Maximilian Wipplinger^1,2^, Parastoo Akbarimoghaddam^2,3,4^, Raquel Alonso-Roman^5^, Zoltan Cseresnyes^3^, Axel Dietschmann^5^, Valentin Wegner^1,2^, Yann Bachelot^2,3,4^, Maria Warschinke ^1,2^Adrian Feile^1,2^, Mohamed I Abdelwahab Hassan^1^, Sonnhild Mittag^1^, Otmar Huber^1^, Bernhard Hube^2,6,7^, Mark S. Gresnigt^2,5^, Marc Thilo Figge^2,3,7^ and Alexander S. Mosig^1,2^ *

* Corresponding author:

E-mail: alexander.mosig@med.uni-jena.de

**Affiliations**

^1^ Institute of Biochemistry II, Jena University Hospital, Jena, Germany

^2^ Cluster of Excellence Balance of the Microverse, Friedrich Schiller University, Jena, Germany

^3^ Applied Systems Biology, Leibniz Institute for Natural Product Research and Infection Biology, Hans Knöll Institute (HKI), Jena, Germany

^4^ Faculty of Biological Sciences, Friedrich Schiller University, Jena, Germany

^5^ Junior Research Group Adaptive Pathogenicity Strategies, Leibniz Institute for Natural Product Research and Infection Biology, Hans Knöll Institute (HKI), Jena, Germany

^6^ Department of Microbial Pathogenicity Mechanisms, Leibniz Institute for Natural Product Research and Infection Biology, Hans Knöll Institute (HKI), Jena, Germany

^7^ Institute of Microbiology, Friedrich Schiller University, Jena, Germany


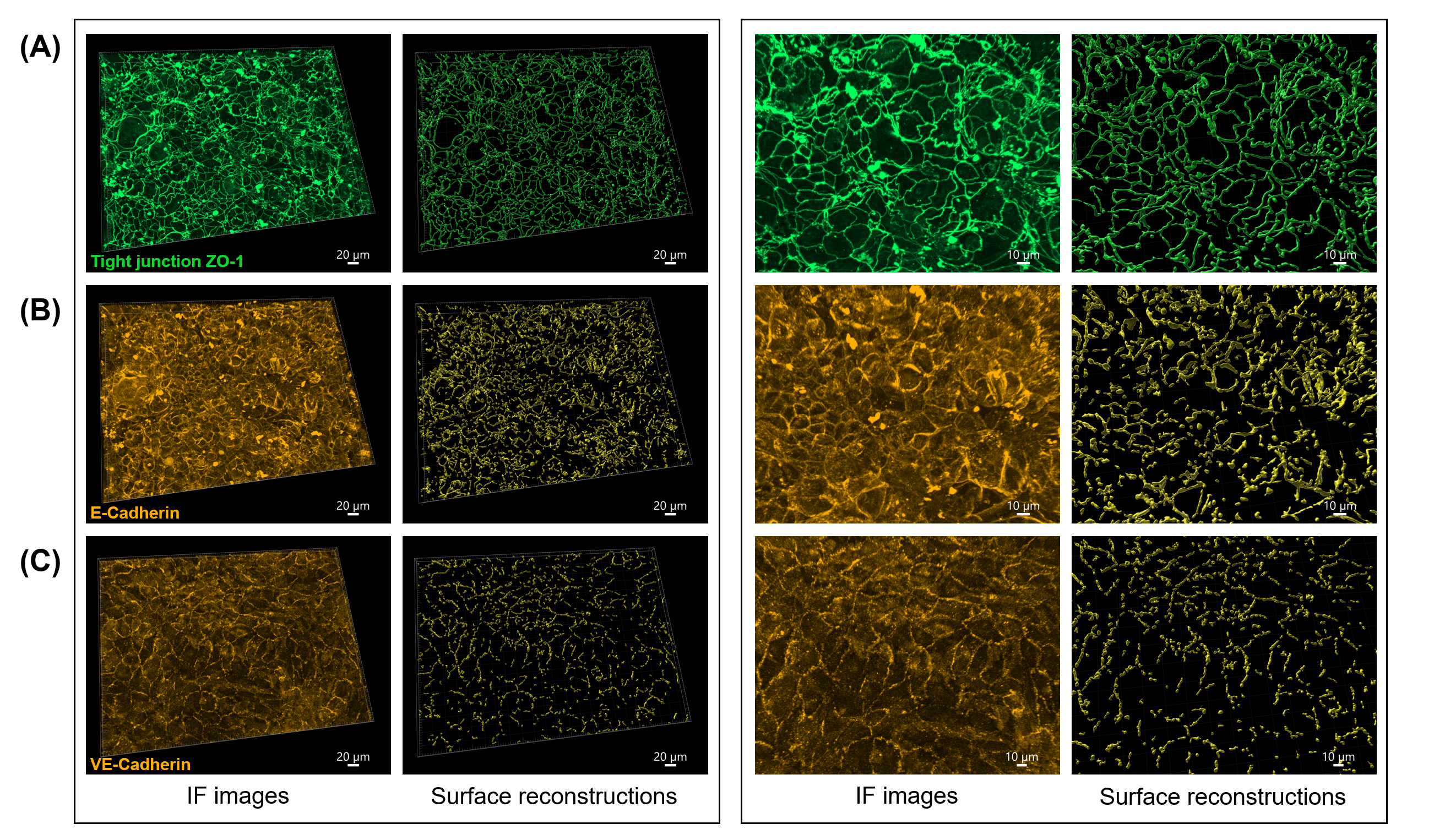


**Supplementary Figure S1.** Segmentation of junctional proteins ZO-1, E-cadherin, and VE-cadherin using IMARIS surface reconstruction. Representative images for ZO-1 **(A)**, E-cadherin **(B)**, and VE-cadherin **(C)**, each displayed in two magnifications; The left panel shows full field-of-view images (scale bar: 20 µm), while the right panel presents corresponding zoomed-in regions (scale bar: 10 µm). Within each panel, raw immunofluorescence images (left) are shown alongside their corresponding 3D surface reconstructions (right), generated using IMARIS. Only segmented surfaces with volumes exceeding 100 voxels were retained to reduce background noise.


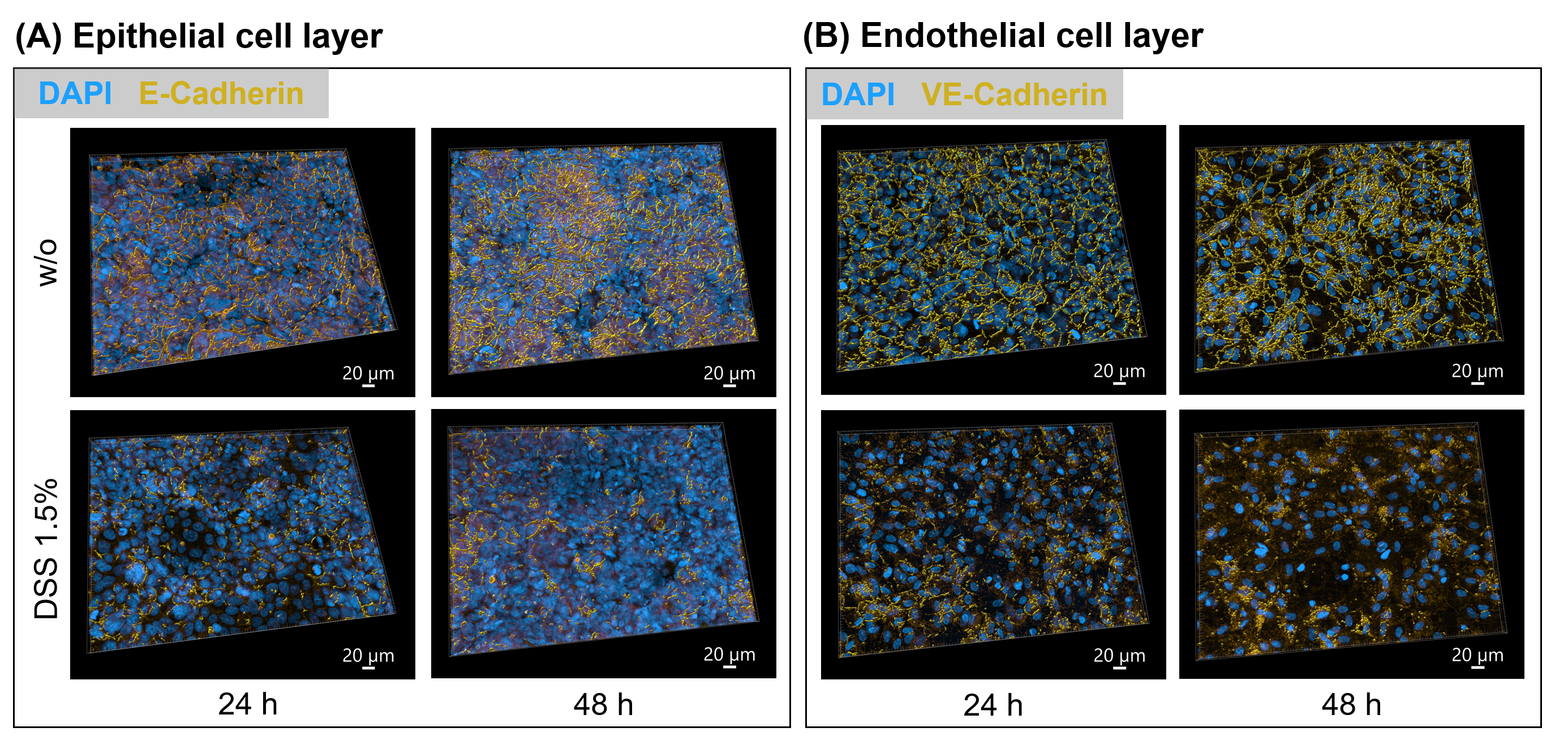


**Supplementary Figure S2. 3D visualization of adherens junction proteins E-cadherin and VE-cadherin under DSS treatment.** Representative 3D reconstructions of **(A)** E-cadherin in the epithelial cell layer and **(B)** VE-cadherin in the endothelial cell layer. For both panels, the upper row shows control samples, and the lower row shows samples treated with 1.5% DSS. Within each row, the left column displays the 24-hour time point and the right column the 48-hour time point. Scale bar: 20 µm.


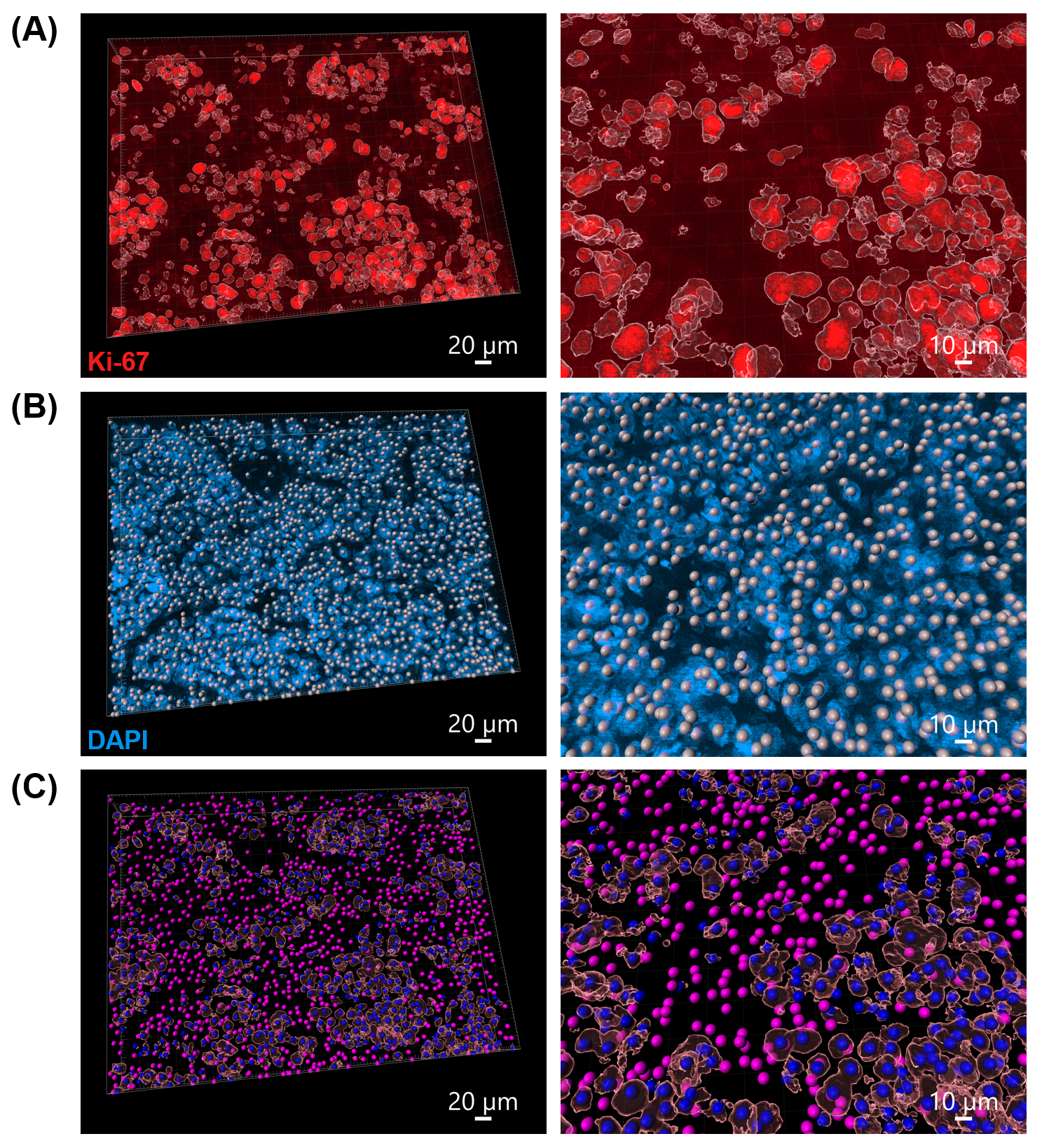


**Supplementary Figure S3. Quantitative analysis of Ki-67⁺ cell detection using IMARIS.** Representative visualization of Ki-67 analysis shown across in full 3D view (left, scale bar: 20 µm), 3D zoomed-in view (right, scale bar: 10 µm). **(A)** Surface reconstruction of the Ki-67 signal. Ki-67 structures are rendered as transparent red surfaces in 3D views. **(B)** Detection of all nuclei using the “Spots” module on the DAPI-stained channel, with an estimated XY diameter of 6 µm. Spots shown as white spheres in 3D. **(C)** Co-localization analysis based on the shortest distance between Ki-67 surfaces and detected nuclei. Nuclei within 1 µm of a Ki-67 surface were classified as Ki-67⁺ (blue), while non-colocalized nuclei were labeled as Ki-67⁻ (magenta).

**
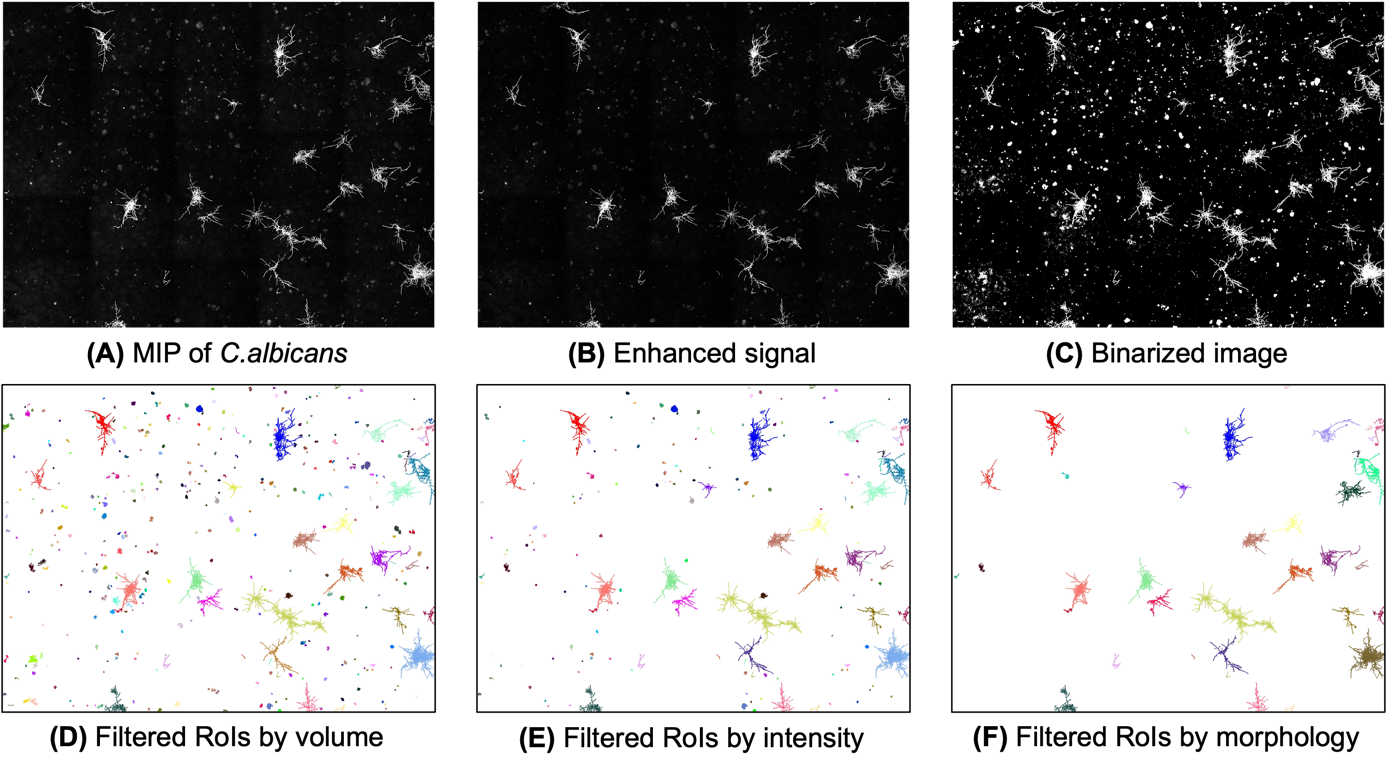
**

**Supplementary Figure S4. Segmentation of *C. albicans* microcolonies.** **A)** Maximum intensity projection (MIP) of the *C. albicans* channel. **B)** Signal enhancement was achieved by applying the rolling ball algorithm, followed by power law intensity transformation. **C)** The image was then binarized using the Triangle thresholding method. **D)** Regions of interest (ROIs) with a volume below 3000 µm³ were removed; although some noise remained, the volume filter was not increased further, in order to avoid losing small microcolonies. **E)** ROIs with average intensity values below 3000, corresponding to background objects, were excluded, which further reduced noise. **F)** Finally, remaining artifacts were removed based on morphology, specifically by excluding ROIs with compactness greater than 0.5, effectively eliminating almost all remaining artifacts.


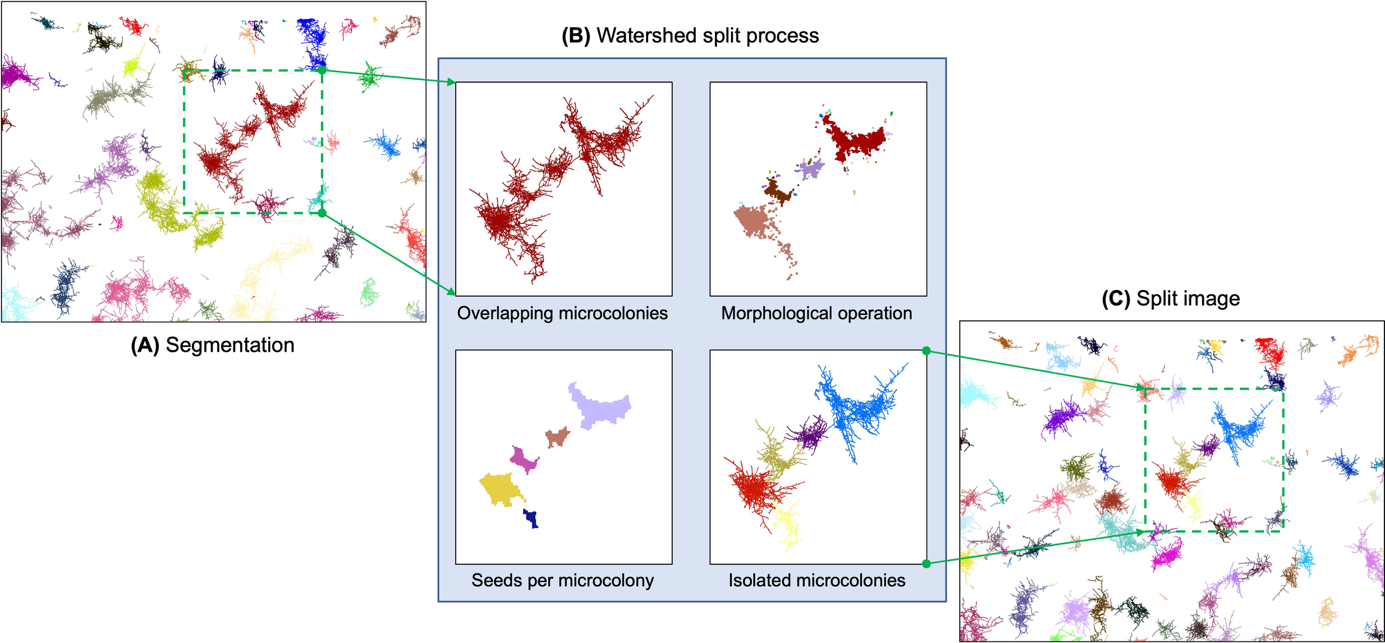


**Supplementary Figure S5.** **3D watershed splitting of *C. albicans* microcolonies. A)** Segmented *C. albicans* microcolonies exhibit significant overlapping. **B)** ROIs with a Feret diameter greater than 500 µm are cropped from the image. Morphological operations, including closing and opening, are applied to remove connecting branches while preserving compact colony centers. An area filter of 1000 µm² and closing is then used to extract the seeds and remove unwanted particles. The watershed algorithm is subsequently applied to isolate individual microcolonies. **C)** The split image is reinserted into its original coordinates, and this process is repeated for all ROIs over 500 µm until all overlapping microcolonies are separated.


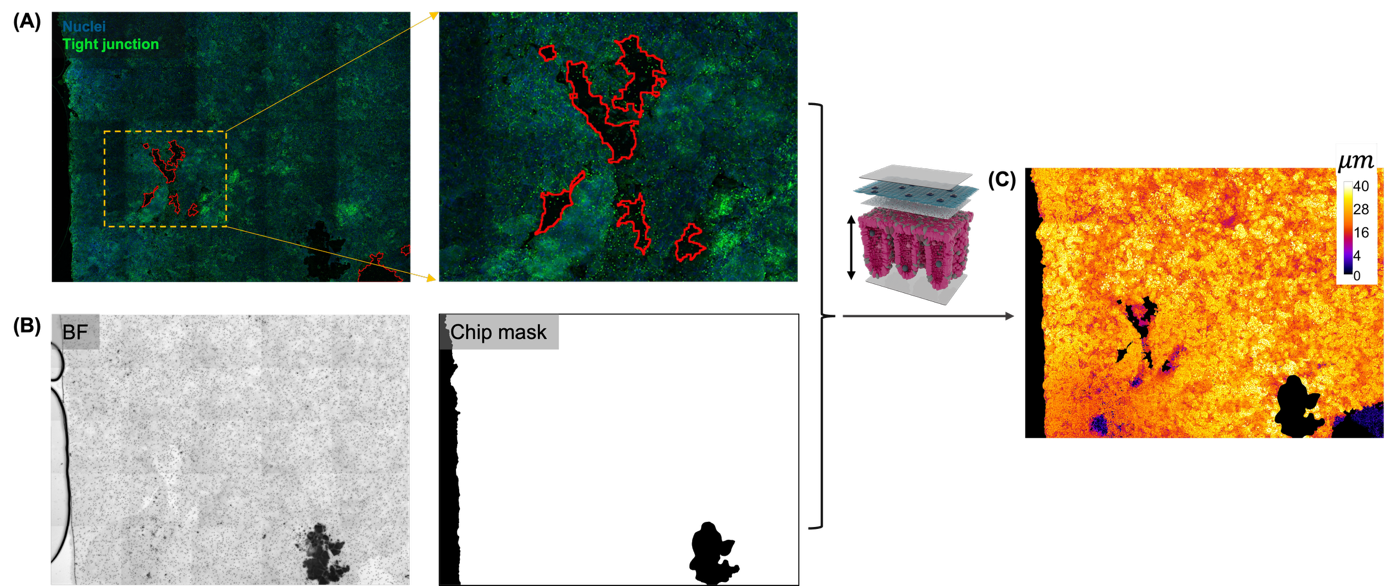


**Supplementary Figure S6. Extraction of pixel-based epithelial tissue thickness map.** **A)** Tissue damage was identified and quantified using a hard threshold approach, with damaged areas marked by a red outline. The damaged sections, highlighted by the dotted yellow bounding box, are displayed as zoomed-in regions on the right. **B)** BF channel highlights that tile scanning can inadvertently capture portions of the membrane edge and artifacts irrelevant to the analysis. A binary mask of the chip area was manually defined to exclude these regions from further analysis. **C)** In this example, the tissue thickness map, representing epithelial tissue thickness at each pixel, ranging from 0 to 40 µm, was generated based on the BF and nuclei channels. Unwanted regions were excluded from the final map, including manually defined membrane edges and automatically segmented tissue damage.


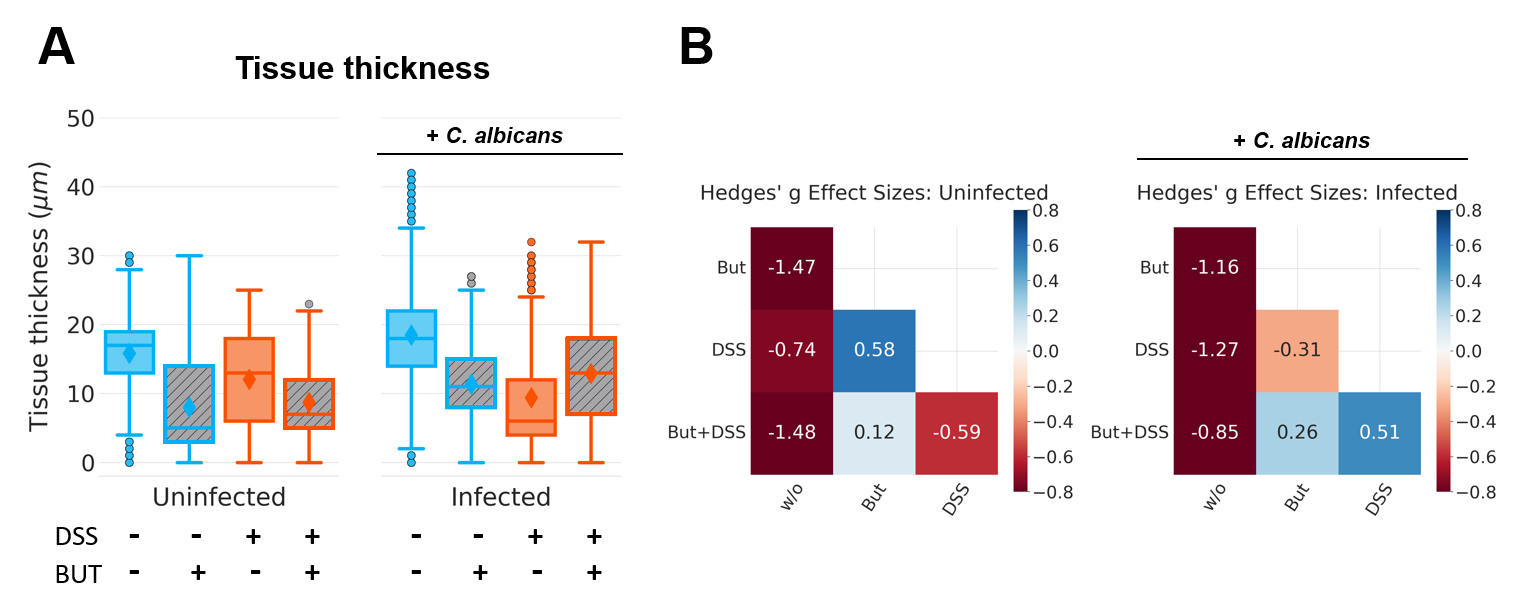


**Supplementary Figure S7. Quantitative analysis of epithelial tissue thickness after DSS and butyrate treatment. A)** Epithelial tissue thickness decreased by DSS treatment both under uninfected and infected conditions, but was only partially restored by butyrate under infected conditions. Box plots represent the 10th – 90th percentile within whisker plots, the box represents the 25th and 75th percentile with the line in the box marking the median and the diamond marking the mean. Data points outside whiskers mark outliers. Thickness distributions were obtained by sampling 10,000 randomly selected points per image, yielding representative thickness profiles for each condition. **B**) Due to the large sample size, effect sizes were calculated using Hedges' g. An effect size of approximately g=0.2 suggests a small effect, of approximately g=0.5 a medium effect, and g≥0.8 signifies a large effect.

**
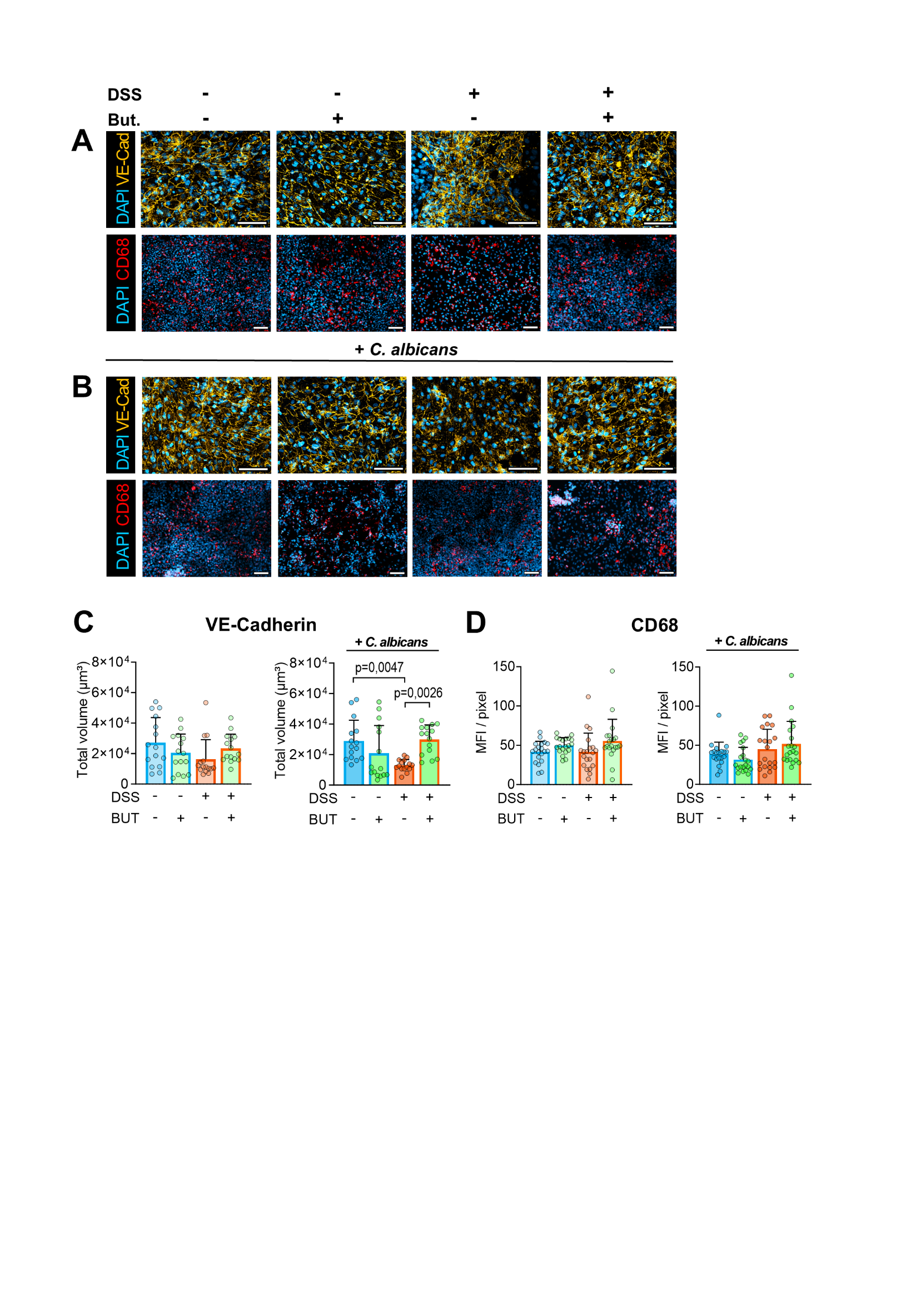
**

**Supplementary Figure S8. Effects of DSS and butyrate on endothelial VE-cadherin structure and macrophage expression level in the colitis-on-chip model. A–B)** Representative immunofluorescence images of endothelial tissue stained for VE-cadherin (yellow), CD68 (red), and DAPI (blue) under **A)** non-infected and **B)** *C. albicans*-infected conditions, with or without DSS (1.5%) and butyrate (1 mM) treatment. Scale bars: 50 µm. **C)** Quantification of 3D junctional VE-cadherin volume reveals a DSS-induced reduction in endothelial junctional integrity under infected conditions, which is restored by butyrate treatment. **D)** Mean fluorescence intensity (MFI) of CD68^+^ macrophages remain stable across all treatment conditions, indicating no significant change in macrophage expression level. Bars represent mean ± SD; individual data points are shown as circles. Statistical analysis was performed using the Kruskal-Wallis test followed by Benjamini-Hochberg corrected Dunn post-hoc comparisons test; exact p-values are indicated.


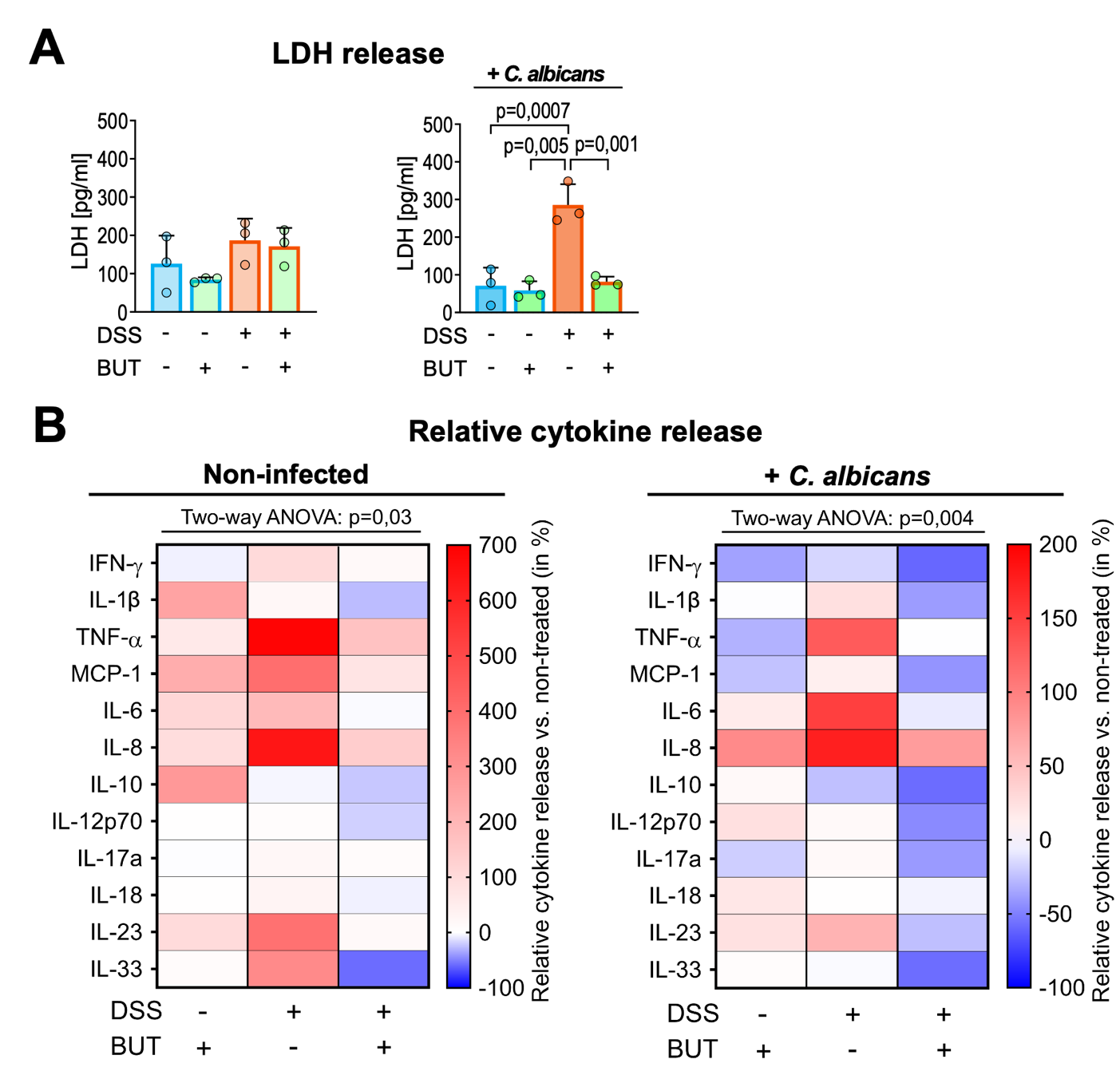


**Supplementary Figure S9. Endothelial cytokine release in the colitis-on-chip model under DSS and butyrate treatment.** Heatmaps showing relative changes in endothelial cytokine release (12 cytokines), normalized to non-treated tissue. Color intensity reflects the percentage change on a dual-gradient scale: -100% (blue) to +700% (red) for non-infected (left), and -100% (blue) to +200% (red) for infected (right). Statistical testing was performed using two-way ANOVA with Tukey’s multiple comparisons test.


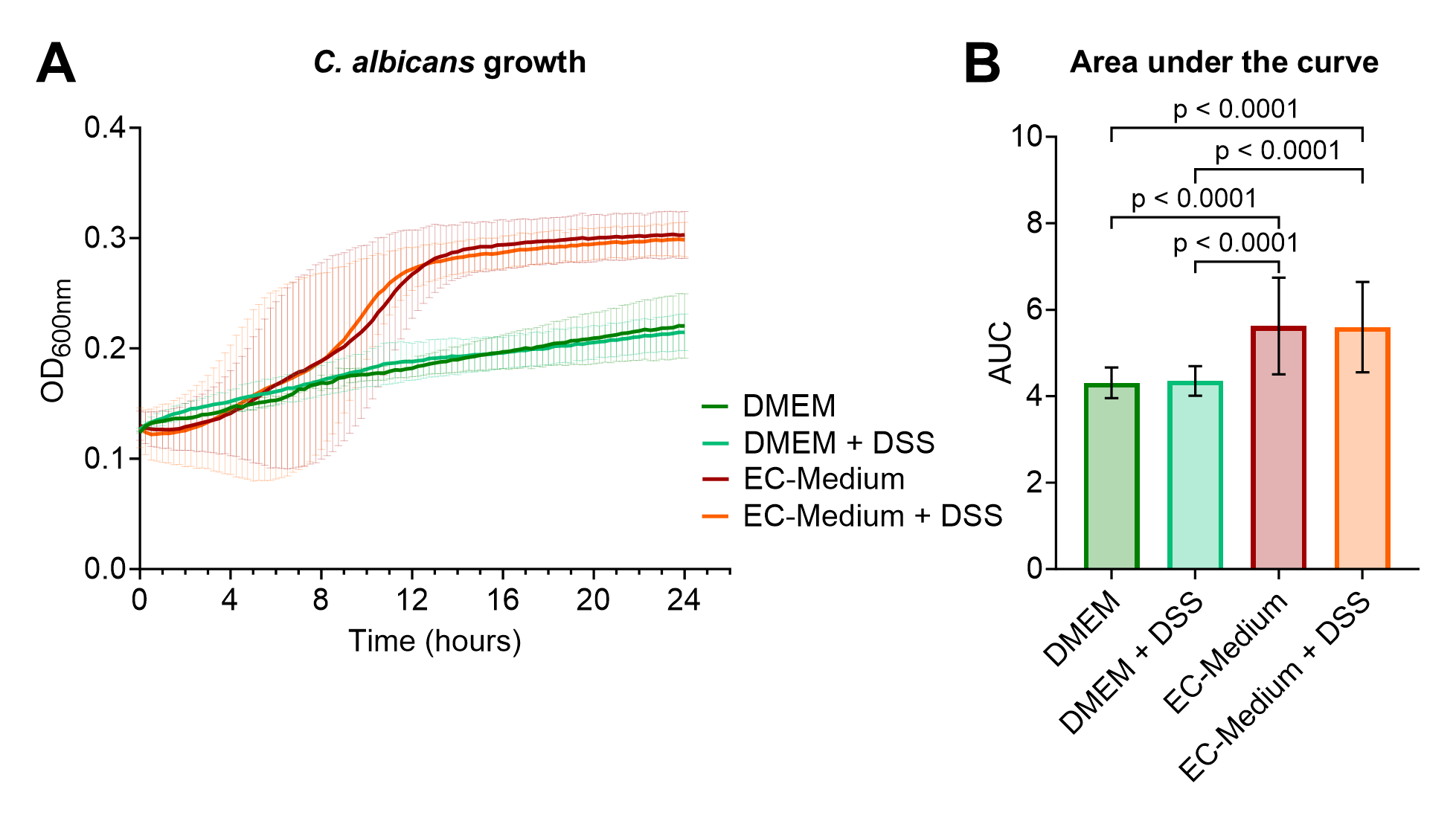


**Supplementary Figure S10. Growth kinetics of *C. albicans* in various cell culture media and under DSS treatment. A)** Growth kinetics of *C. albicans* over 24 hours in DMEM or EC-Medium, with or without adding 1.5% DSS. Optical density at 600 nm (OD600) was measured every 15 minutes. Data are shown as mean ± SD. **B)** Area under the curve (AUC) analysis of the growth curves shown in (A), demonstrating significantly enhanced fungal growth in EC-Medium compared to DMEM, with further increases upon DSS addition. Statistical comparisons were performed using unpaired two-tailed t-tests; exact p-values are indicated.


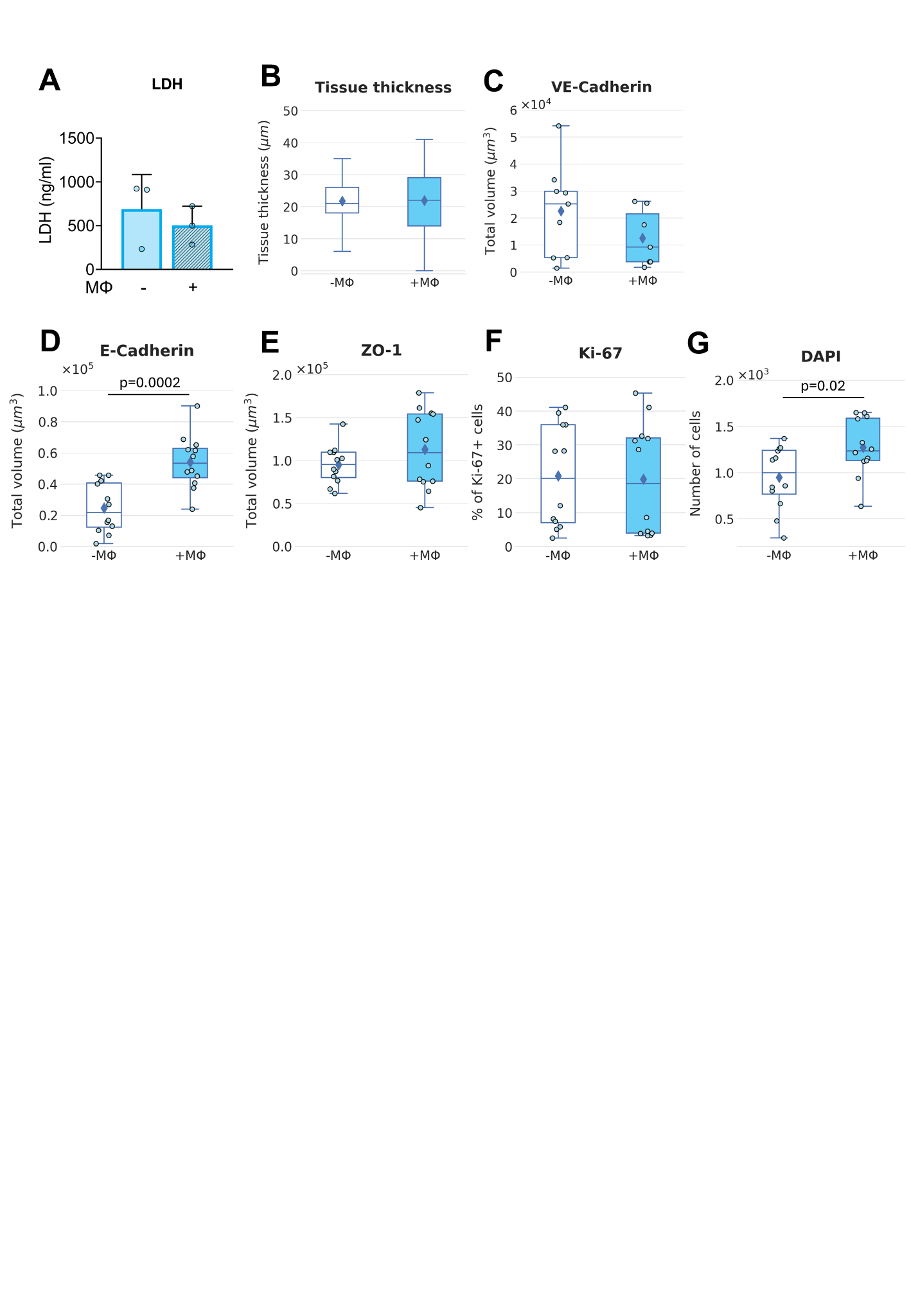


**Supplementary Figure S11. Effect of macrophages on baseline tissue parameters in the intestine-on-chip model.** Quantification of **A)** LDH release from the endothelial side, **B)** epithelial tissue thickness, **C)** total 3D junctional VE-cadherin volume, **D)** total 3D junctional E-cadherin volume, **E)** total 3D junctional ZO-1 volume, **F)** percentage of Ki-67-positive epithelial cells, and **G)** total number of DAPI^+^  cells. All analyses were performed under untreated baseline conditions, comparing chips with (striped, filled blue) and without (solid, unfilled) integrated macrophages. Bars represent mean ± SD; box plots with whisker plots showing the 0th – 100th percentile, the box represents the 25th and 75th percentile, with the line in the box marking the median and the diamond marking the mean. Individual values are shown as circles. Statistical analysis was performed using a two-tailed t-test each.


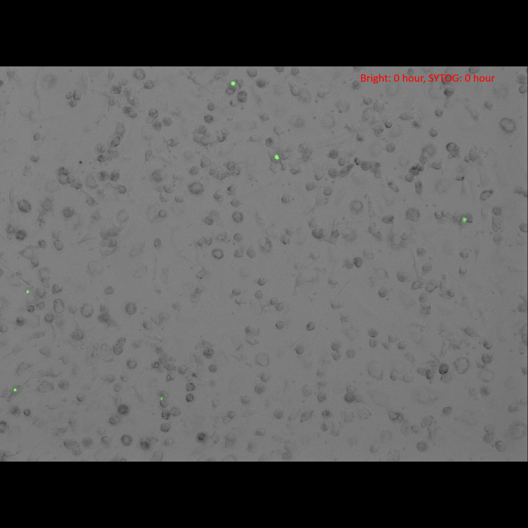

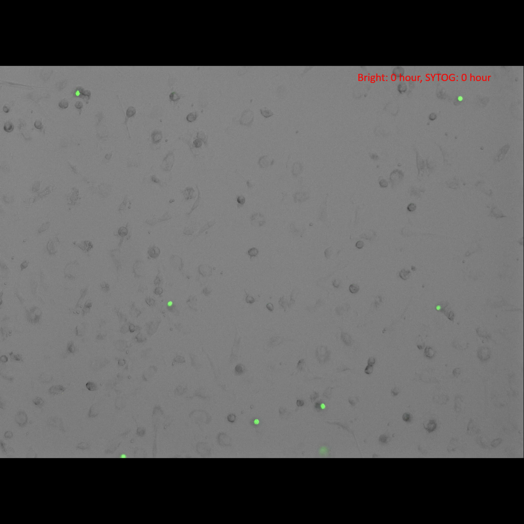


**Supplementary Video 1. Time-lapse imaging of macrophage viability during *C. albicans* infection with and without butyrate pre-treatment.** Live-cell imaging over 24 h showing SYTOX-Green-stained macrophages in co-culture with *C. albicans*. Left: untreated macrophages; right: macrophages pre-incubated with 1 mM butyrate for 24 h. Green fluorescence indicates dead cells. Butyrate pre-treatment significantly delayed the onset of macrophage death and improved overall cell viability during fungal infection.


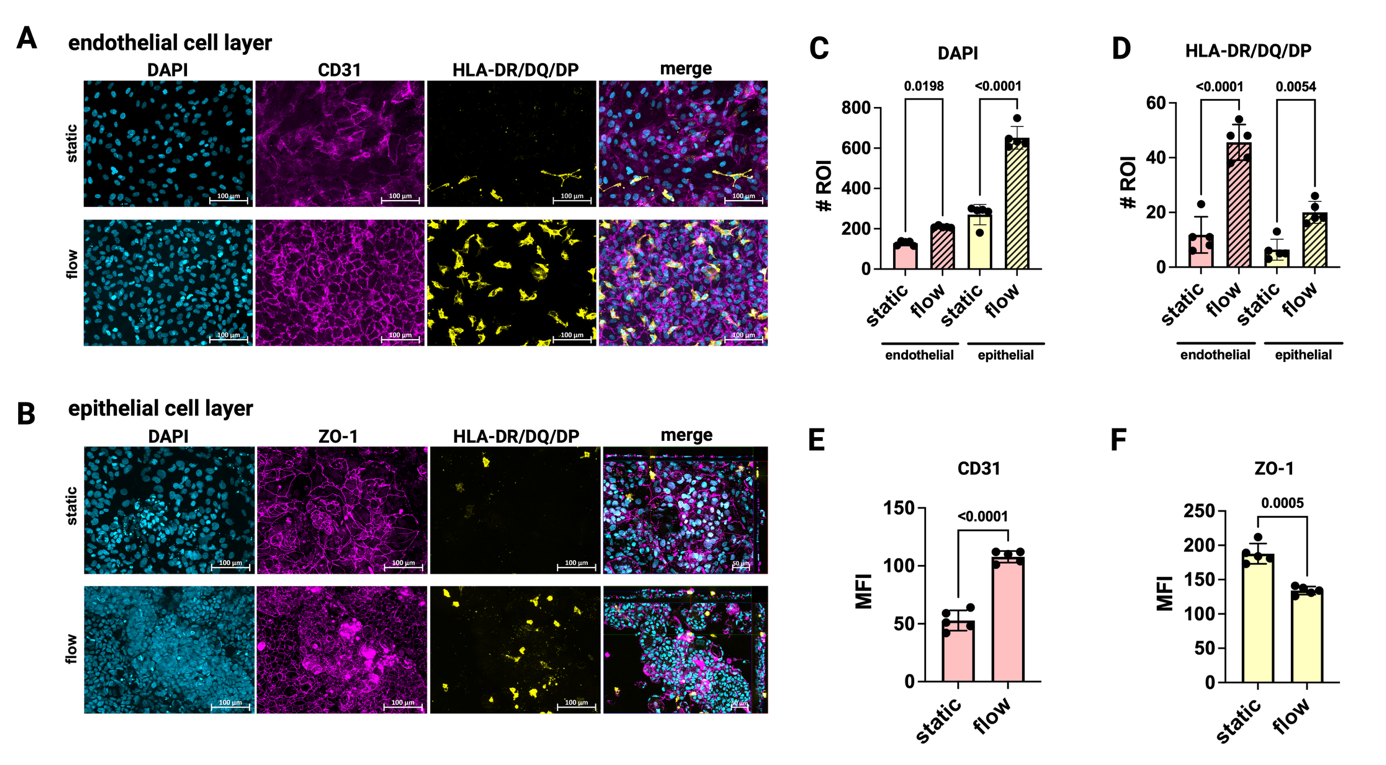


**Supplementary Figure S12. Effect of continuous perfusion on endothelial and epithelial tissue organisation and macrophage integration in the immunocompetent gut-on-chip model. A)** Immunofluorescence images of the endothelial compartment under static (top) and flow (bottom) conditions. DAPI (nuclei, blue), CD31 (magenta), HLA-DR/DQ/DP (green), and merged image. **B)** Immunofluorescence images of the epithelial compartment under static (top) and flow (bottom) conditions. DAPI (nuclei, blue), ZO-1 (magenta), HLA-DR/DQ/DP (green), and merged image. A-B) Scale bars, 100 µm. **C-D)** Quantification of endothelial and epithelial marker expression as the number of segmented objects (#ROI) for **C)** DAPI (nuclei) and **D)** HLA-DR/DQ/DP (macrophages). **E-F)** Quantification of expression of endothelial marker E) CD31 and epithelial marker **F)** ZO-1 as mean fluorescence intensity (MFI, right) under static and flow conditions. **C-F)** Results are shown for static (open bars) and flow (hatched bars) conditions. Data from three independent experiments are shown. Bars represent mean ± SD; individual data points are shown as circles.


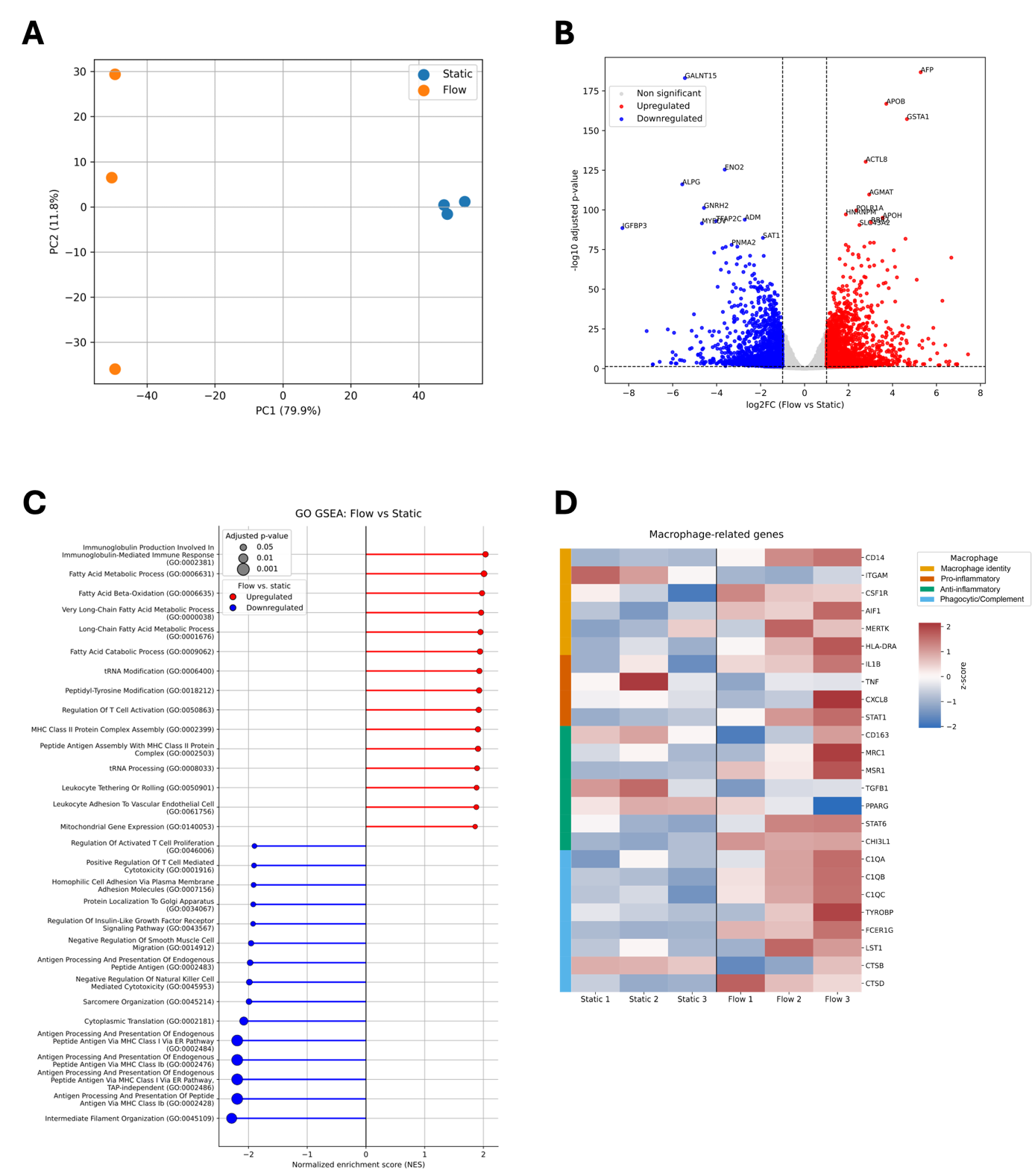


**Supplementary Figure S13.** **Bulk RNA-sequencing reveals flow-dependent transcriptomic reprogramming in the intestine-on-chip model.** **A)** Principal component analysis (PCA) of bulk RNA-seq samples cultured under static and flow conditions. PCA was performed on log2-transformed counts per million (log2-CPM) values. Biological replicates clustered closely within each condition, and static and flow samples separated along the first principal component axis PC1, indicating condition-dependent transcriptomic differences. Flow conditions introduced more variability than static culture, as visible by the spread of biological replicates along PC2. **B)** Volcano plot showing differential gene expression between flow and static conditions. Each dot represents one gene. Genes with adjusted p-value < 0.05 and absolute log2 fold change > 1 were classified as significantly differentially expressed and are highlighted according to direction of change. Of 18,613 genes analyzed, 2,192 were significantly upregulated (red) and 1,966 were significantly downregulated (blue) under flow. **C)** Gene set enrichment analysis (GSEA) of ranked differential expression results. The normalized enrichment score (NES) indicates the direction and magnitude of pathway enrichment. Positive NES values (red) indicate gene sets enriched under flow, negative NES values (blue) indicate gene sets enriched under static conditions. Dot size reflects statistical significance (adjusted p-value). Flow conditions were associated with enrichment of immune activation pathways including MHC class II assembly, leukocyte adhesion, and T cell regulation, as well as fatty acid metabolic and mitochondrial gene expression programs. Static conditions showed enrichment of MHC class I antigen processing and intermediate filament organization pathways. **D)** Heatmap of curated macrophage-associated marker genes showing row-wise z-score-transformed log2-CPM values. Genes were grouped into macrophage identity, pro-inflammatory, anti-inflammatory, and phagocytic/complement-associated categories, indicated by the row colour annotation. Columns represent individual biological replicates (n = 3 per condition). Flow conditions promoted coordinated upregulation across all macrophage-functional categories, indicating maturation toward a tissue-resident macrophage phenotype under perfusion.

**Overview of Normality Assessment and Statistical Test Selection**

Normality was assessed for all datasets **with more than 10 data points** per condition using both **quantile-quantile (Q-Q) plots** and the **Shapiro-Wilk test**. In the Q-Q plots, data points that closely follow the diagonal reference line indicate that the dataset is approximately normally distributed. Consistently, a **Shapiro-Wilk p-value greater than 0.05** suggests that the data do not significantly deviate from a normal distribution. For smaller sample sizes (less than 10 **data points** per condition), **parametric statistical tests** **such as** unpaired t-tests, one-way and two-way ANOVA with multiple comparisons were used, as non-parametric methods lack sufficient statistical power in such cases.

The following figure summarizes the results of the normality assessments for **Figure 1, Figure 3, Supplementary Figure S8, and Supplementary Figure S11**. Based on these assessments, **parametric tests** were applied to the datasets in **Figure 1** and **Supplementary Fig. S11**, which largely followed a normal distribution. In contrast, the datasets in **Figure 3** and **Supplementary Fig. S8**, which did not meet normality assumptions, were analyzed using the **non-parametric Kruskal–Wallis test** followed by **Benjamini–Hochberg–corrected Dunn post-hoc comparisons**.


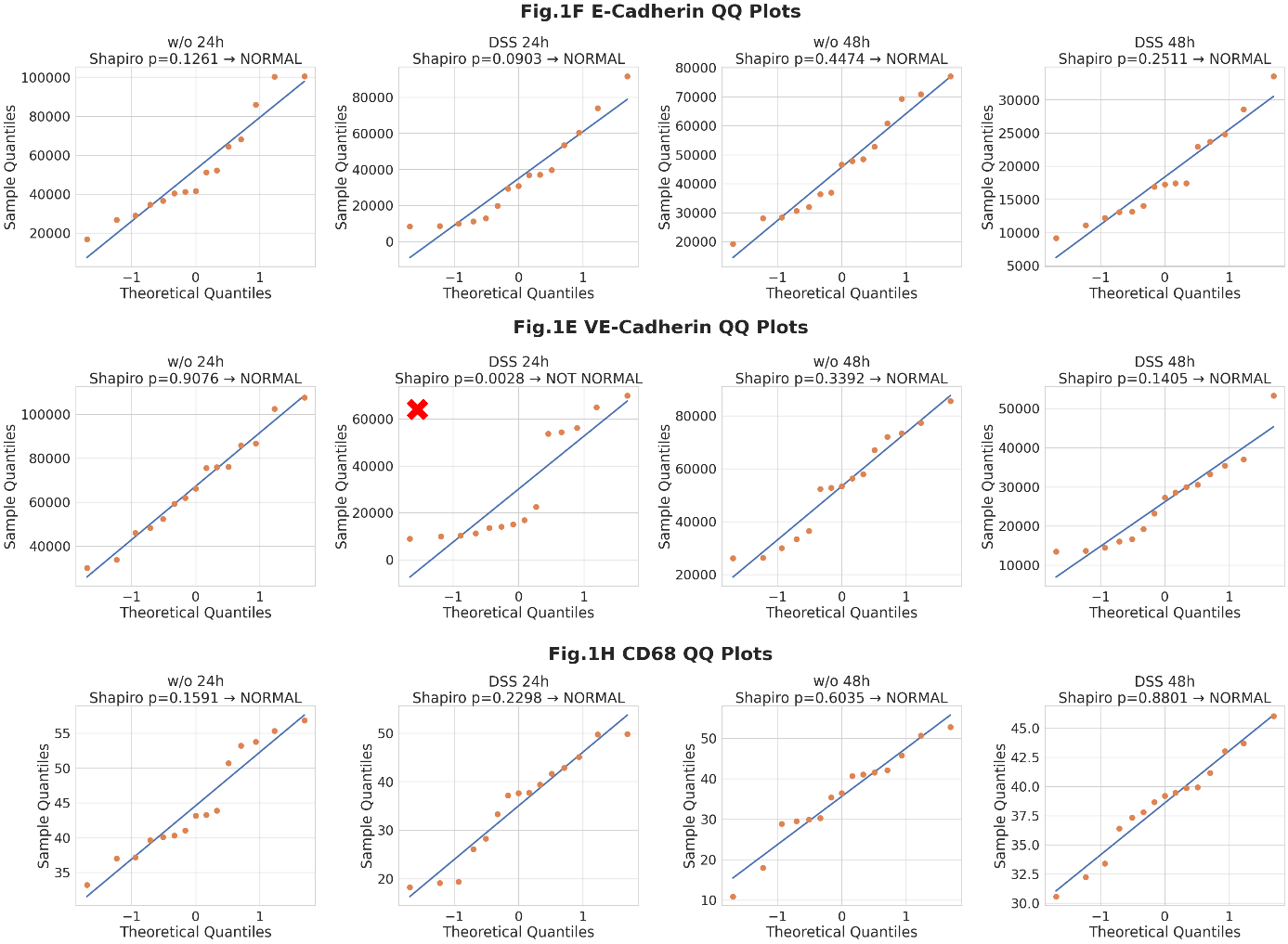


**Supplementary Figure S14. Normality assessment for datasets displayed in Figure 1.** QQ plots evaluating the distribution of **E-Cadherin**, **VE-Cadherin**, and **CD68** quantitative values under four experimental treatments (**w/o 24h**, **DSS 24h**, **w/o 48h**, **DSS 48h**). Each subplot shows theoretical versus sample quantiles with a fitted reference line. **Shapiro-Wilk normality test results** (p-values and interpretation) are reported above each panel. A **red cross,** marks datasets that significantly deviate from normality (**Shapiro-Wilk** p < 0.05).

**
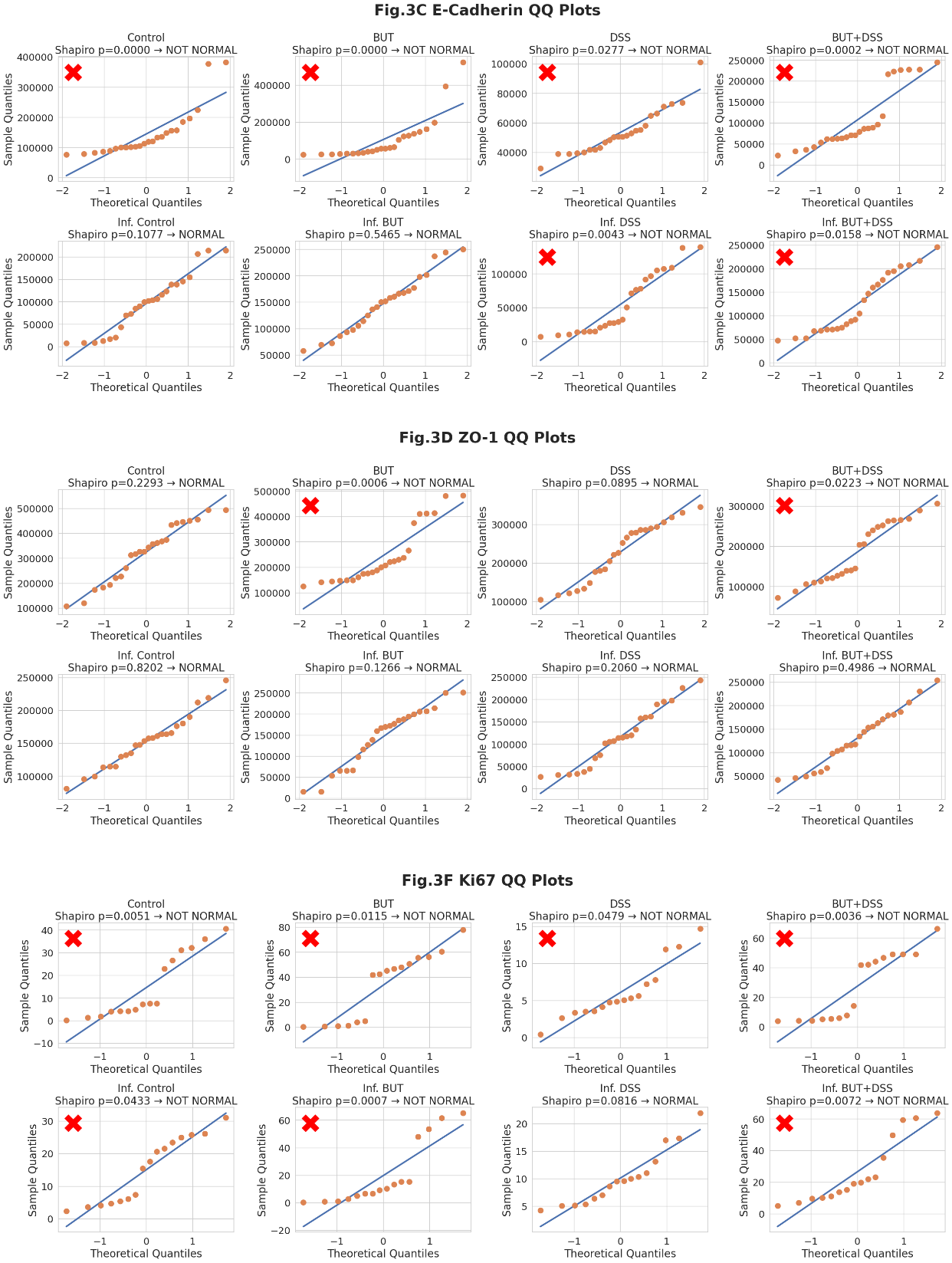
**

**Supplementary Figure S15. Normality assessment for datasets displayed in Figure 3.** QQ plots evaluating the distribution of **E-Cadherin**, **ZO-1**, and **Ki67** quantitative values under eight experimental treatments (Uninfected samples: Control, BUT, DSS, BUT+DSS and infected samples: Inf. Control, Inf. BUT, Inf. DSS, Inf. BUT+DSS). Each subplot shows theoretical versus sample quantiles with a fitted reference line. **Shapiro-Wilk normality test results** (p-values and interpretation) are reported above each panel. A **red cross,** marks datasets that significantly deviate from normality (**Shapiro-Wilk** p < 0.05).


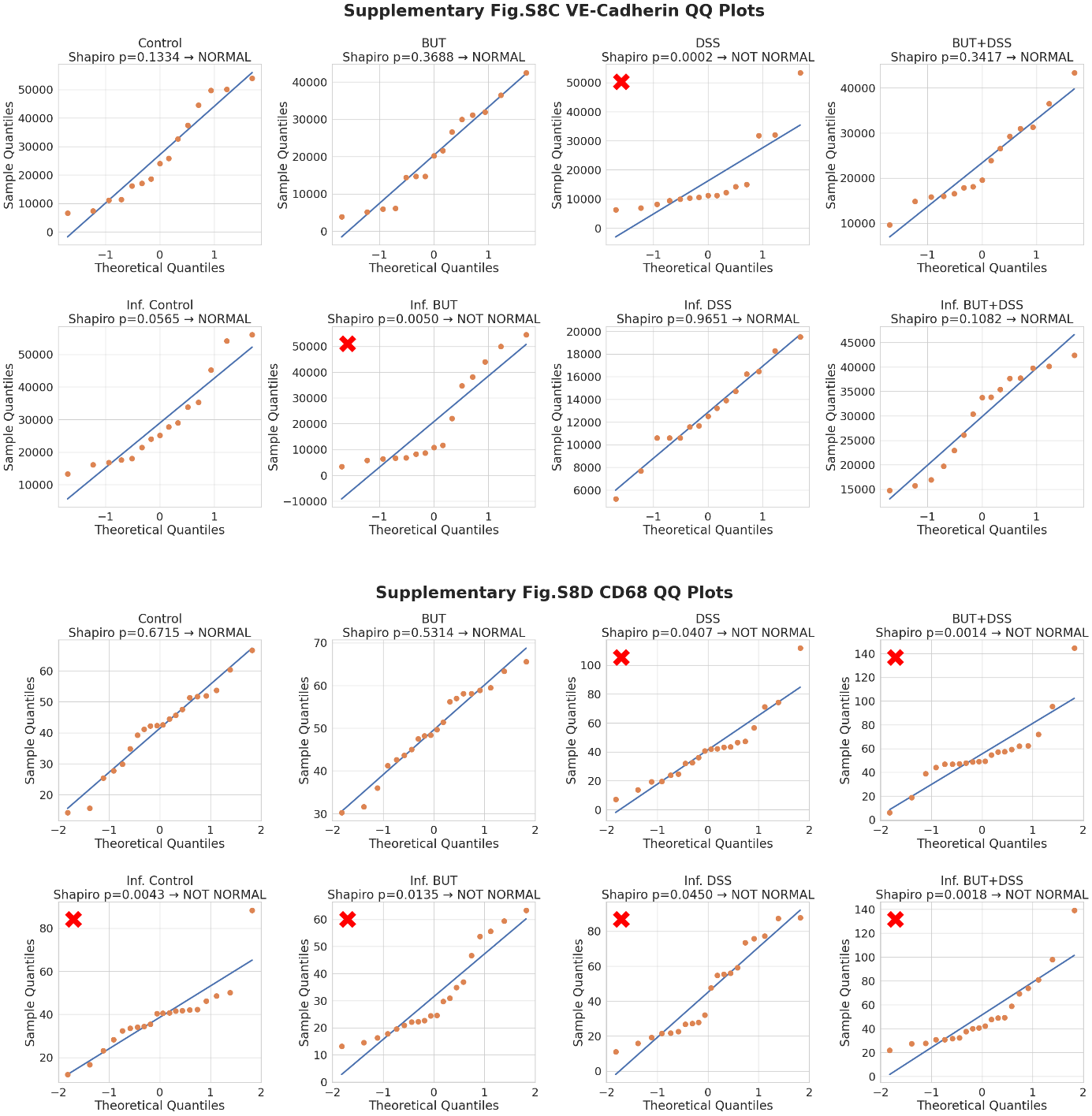


**Supplementary Figure S16. Normality assessment for datasets displayed in Supplementary Figure S8.** QQ plots evaluating the distribution of V**E-Cadherin and** CD68 quantitative values under eight experimental treatments (Uninfected samples: Control, BUT, DSS, BUT+DSS and infected samples: Inf. Control, Inf. BUT, Inf. DSS, Inf. BUT+DSS). Each subplot shows theoretical versus sample quantiles with a fitted reference line. **Shapiro-Wilk normality test results** (p-values and interpretation) are reported above each panel. A **red cross,** marks datasets that significantly deviate from normality (**Shapiro-Wilk** p < 0.05).


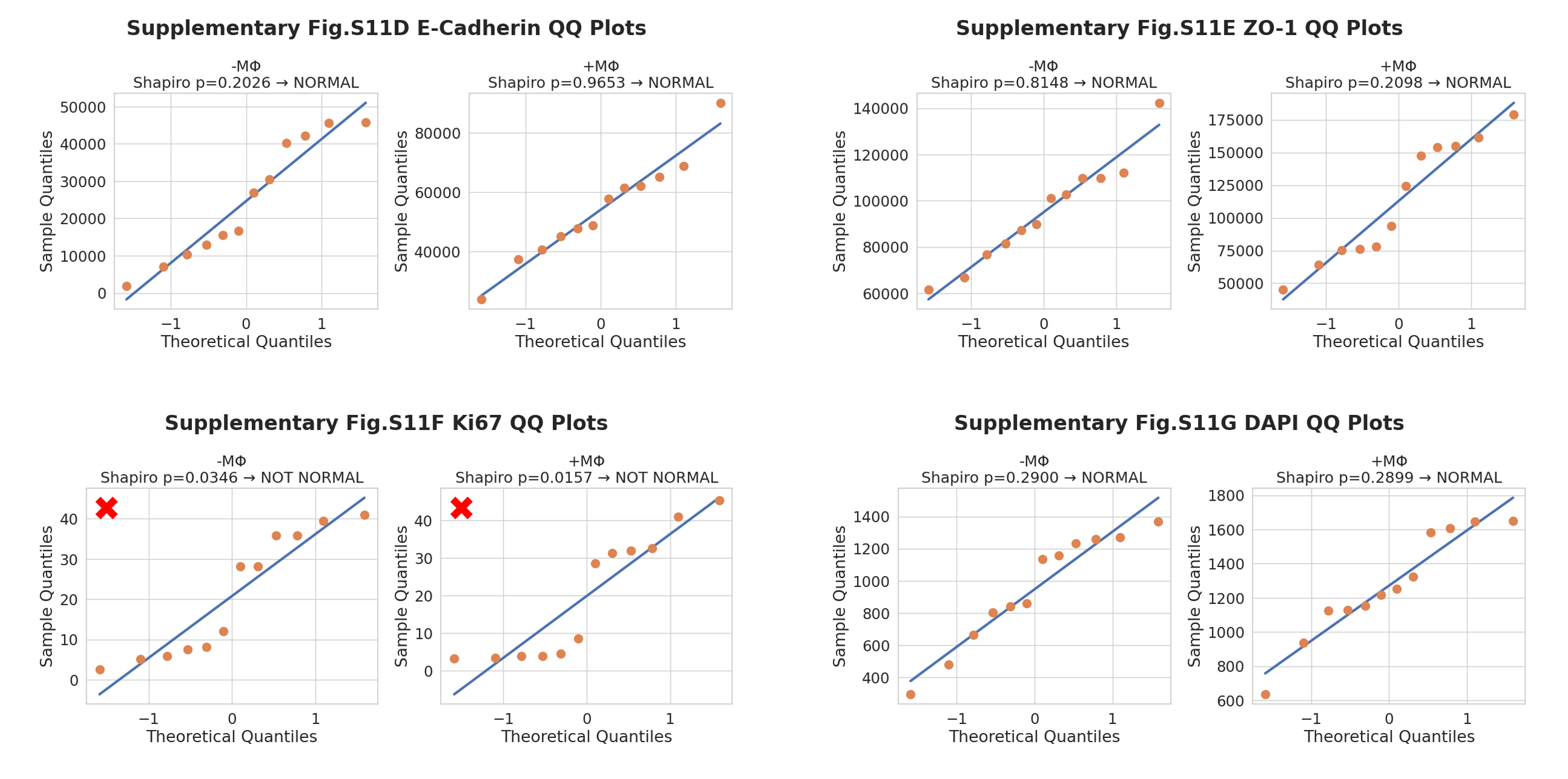


**Supplementary Figure S17. Normality assessment for datasets displayed in Supplementary Figure S11.** QQ plots evaluating the distribution of **E-Cadherin, ZO-1, Ki67 and DAPI** quantitative values under two experimental treatments (without and with macrophages). Each subplot shows theoretical versus sample quantiles with a fitted reference line. **Shapiro-Wilk normality test results** (p-values and interpretation) are reported above each panel. A **red cross,** marks datasets that significantly deviate from normality (**Shapiro-Wilk** p < 0.05).
